# Ancestrality and evolution of trait syndromes in finches (Fringillidae)

**DOI:** 10.1002/ece3.3420

**Published:** 2017-10-21

**Authors:** Jean‐François Ponge, Dario Zuccon, Marianne Elias, Sandrine Pavoine, Pierre‐Yves Henry, Marc Théry, Éric Guilbert

**Affiliations:** ^1^ Muséum National d'Histoire Naturelle Mécanismes adaptatifs et évolution (MECADEV UMR 7179) Sorbonne Universités, MNHN, CNRS Brunoy France; ^2^ Muséum National d'Histoire Naturelle Service de Systématique Moléculaire Paris France; ^3^ Muséum National d'Histoire naturelle Institut de Systématique, Évolution, Biodiversité (ISYEB UMR 7205) CNRS, MNHN, UPMC, EPHE Sorbonne Universités Paris France; ^4^ Muséum National d'Histoire Naturelle Centre d’Écologie et des Sciences de la Conservation (CESCO UMR 7204) MNHN, CNRS, UPMC Sorbonne Universités Paris France

**Keywords:** ancestral state, evolution, Fringillidae, phylogeny, phylogeography, strategies

## Abstract

Species traits have been hypothesized by one of us (Ponge, 2013) to evolve in a correlated manner as species colonize stable, undisturbed habitats, shifting from “ancestral” to “derived” strategies. We predicted that generalism, r‐selection, sexual monomorphism, and migration/gregariousness are the ancestral states (collectively called strategy A) and evolved correlatively toward specialism, K‐selection, sexual dimorphism, and residence/territoriality as habitat stabilized (collectively called B strategy). We analyzed the correlated evolution of four syndromes, summarizing the covariation between 53 traits, respectively, involved in ecological specialization, r‐K gradient, sexual selection, and dispersal/social behaviors in 81 species representative of Fringillidae, a bird family with available natural history information and that shows variability for all these traits. The ancestrality of strategy A was supported for three of the four syndromes, the ancestrality of generalism having a weaker support, except for the core group Carduelinae (69 species). It appeared that two different B‐strategies evolved from the ancestral state A, both associated with highly predictable environments: one in poorly seasonal environments, called B1, with species living permanently in lowland tropics, with “slow pace of life” and weak sexual dimorphism, and one in highly seasonal environments, called B2, with species breeding out‐of‐the‐tropics, migratory, with a “fast pace of life” and high sexual dimorphism.

## 1 INTRODUCTION

Ponge (2013) suggested that a wide array of traits related to ecological niche requirements (e.g., specialist vs. generalist), life history (e.g., K‐ vs. r‐selection), behavior (e.g., residents vs. dispersers, territorial vs. gregarious), and selection mode (e.g., sexual vs. natural selection) co‐evolved along gradients of environmental predictability, forming two suites of generalized syndromes or evolutionary strategies. According to this theory, the ancestral suite of syndromes, here collectively called strategy A and previously called “barbarian” strategy in Ponge (2013), includes generalism, r‐selected traits, dispersiveness/gregariousness, and more generally traits under natural selection (as opposed to sexual/social selection). It is associated with disturbed and unpredictable environments (Sallan & Galimberti, 2015; Sheldon, 1996). Conversely, opposite trait modalities (values taken by a given trait under selection) include specialism, K‐selected traits, residence/territoriality, and traits under sexual/social selection, here collectively called strategy B and previously called “civilized” strategy in Ponge (2013). They are predicted to evolve in stable environments with a high level of exploitative competition through character displacement (Brown & Wilson, 1956) and convergence (Laiolo et al., 2015), allowing species to segregate and become finely tuned to their environment. Given the number of evolutionary steps necessary for being finely tuned to the environment (Poisot, Bever, Nemri, Thrall, & Hochberg, 2011), trait modalities of strategy B would thus be in derived positions in phylogenetic trees (see Raia, Carotenuto, Passaro, Fulgione, & Fortelius, 2012; for a discussion about the derived position of ecological specialization). Conversely, trait modalities of strategy A would thus be in ancestral position along phylogenetic trees. This suggests a macroevolutionary trade‐off between the need to move and/or reproduce and the need to specialize to stable resources and habitats (see Berg et al., 2010; Bonte et al., 2012; Rottenberg, 2007). Tropical lowland areas, in particular tropical rainforests, known for their greater stability and lower energetic cost for organisms compared to areas with seasonal stress (Janzen, 1967), are thus expected to harbor more B‐strategy traits, thought to be derived (Cardillo, 2002).

In this article, we want to test simultaneously, for the first time, in a monophyletic group, the following hypotheses: generalism, r‐selection, natural selection, and dispersiveness/gregariousness (strategy A) are ancestral and tend to shift toward derived (strategy B) attributes in the course of evolution (Hypothesis 1), and these four trait syndromes evolve correlatively (Hypothesis 2). At last, we hypothesize that state B traits are favored by two kinds of predictable environments: stable and benign tropical environments favor state B traits only, while stable but seasonal or harsh environments favor a combination of state A and state B traits (Hypothesis 3). To test these hypotheses, we chose to work with birds because their traits are particularly well‐documented, thanks to a long tradition of natural history documentation by ornithologists (Schmeller, Henle, Loyau, Besnard, & Henry, 2012). We selected the bird family Fringillidae (i.e., true finches) because (1) it is distributed worldwide, (2) it is known for its wide range of variation in terms of ecological specialization, life history, social/dispersal behavior, and sexual dimorphism (Clement, Harris, & Davis, 2011), and (3) its phylogeny is well‐established on the basis of representatives of all existing lineages (Zuccon, Prŷs‐Jones, Rasmussen, & Ericson, 2012). This bird family is a good model to follow the evolution of multiple suites of traits, as attested by several studies performed on cardueline finches (Badyaev, 1997a,b,c; Badyaev & Ghalambor, 1998; Badyaev, Hill, & Weckworth, 2002).

## 2 MATERIAL AND METHODS

### 2.1 Data collection and preparation

The literature on Fringillidae (153 references, Appendix S1) was used to collect the necessary natural history data (Appendix S2). Fifty‐three variables were documented (Appendix S3). For each quantitative variable (measurements, count data) and each species, we used the weighted arithmetic mean value across all available data. Qualitative data (coded as “Yes” or “Yes minus” in Appendix S2) were coded as 1 for the presence of the character, 0 for its absence, 0.5 for its partial presence). When the same character exhibited different trait modalities (e.g., foraging height), data were scored according to an arbitrary scale (e.g., foraging height was scored 1 for ground, 2 for shrubs, 3 for trees). The variance of these numerical values (weighted by their occurrence in literature) was complemented to 1 and was used as synthetic index of specialization (e.g., foraging height specialization in Appendix S3). Altitudinal specialization of a given species was measured by dividing its altitudinal range (maximum minus minimum altitude) by the maximum altitude found in our dataset (i.e., 4,950 m) and complementing to 1 this ratio. The same method based on weighted occurrence was used to measure the intra‐specific variability of a behavioral trait when it exhibited different trait modalities (e.g., breeding dispersion and migration). Missing data (20 ± 23% of total records, Appendix S3) were interpolated according to a nearest‐neighbor method (Holmes & Adams, 2002) prior to Principal Component Analysis (PCA).

### 2.2 Phylogenetic tree

The Fringillidae phylogeny by Zuccon et al. (2012) included 93 ingroup taxa of 205 extant species (sensu Dickinson & Christidis, 2014), representing all major lineages, genera, and species groups and was based on a combination of three nuclear and two mitochondrial genes. Here, we used the substitution rates for a clade of Fringillidae, the drepanids, obtained by Lerner, Meyer, James, Hofreiter, and Fleischer (2011), to time‐calibrate the phylogeny of Fringillidae. Because 12 species had <50% of scored ecological traits, they were excluded from the analysis. The time‐calibrated phylogeny was generated with BEAST 1.8.0 (Drummond & Rambaut, 2007), as implemented in the CIPRES Science Gateway (Miller, Pfeiffer, & Schwartz, 2010). We assumed an uncorrelated molecular clock model, a speciation yule tree prior and a GTR+Γ or GTR+Γ+I substitution model (Posada & Crandall, 2001), for nuclear or mitochondrial genes, respectively. Markov chain Monte Carlo (MCMC) simulations were run for 100 million generations with sampling every 10,000 generations. The convergence was evaluated in Tracer 1.6, and the maximum clade credibility tree was summarized using TreeAnnotator v1.8.0 (both packages implemented in BEAST), excluding the first 25% trees as burn‐in.

This phylogeny was used for the reconstruction of ancestral syndromes, for the phylogenetic Principal Component Analysis (pPCA) and for modeling correlations between transitions from ancestral to derived states, as explained in the following sections. The tree that we obtained covers about 40% of Fringillidae diversity. No global phylogeny for the entire family is available, but a number of complete or almost complete phylogenies have been published for some subclades/genera, for example, *Pyrrhula* (Töpfer et al., 2011), *Carpodacus* sensu lato (Tietze, Päckert, Martens, Lehmann, & Sun, 2013), *Haemorhous* (Smith, Bryson, Chua, Africa, & Klicka, 2013), and *Spinus* (Beckman & Witt, 2015). We evaluated the representativeness of the family diversity in our phylogeny by a qualitative comparison against a supermatrix tree. Using the dataset used by Zuccon et al. (2012) as starting point, we assembled a supermatrix for three nuclear introns and five mitochondrial genes by comparison with the datasets of other published phylogenies and searching in Genbank for any additional finch sequence (Appendix S4). The supermatrix tree was estimated by maximum likelihood using RAxML version 7.0.3 (Stamatakis, 2006), applying a gene partition, a GTR+Γ+I model, and random starting tree, with α‐shape parameters, GTR‐rates, and empirical base frequencies estimated and optimized for each partition. Nodal support was estimated using 100 bootstrap replicates.

We calculated phylogenetic distances between two species in the supermatrix tree as the sum of branch lengths on the smallest path that connects the two species. Next we calculated the mean phylogenetic distance (MPD) between two species among the 81 species selected for our analysis. We analyzed whether the selected species were more phylogenetically clustered than expected randomly using a null model. We simulated 1,000 null communities by selecting randomly 81 species in the supermatrix tree (outgroup excluded). The *p*‐value (quantile) was calculated as the proportion of the MPD of the null communities that were lower than the observed MPD. We obtained observed MPD = 0.319 and *p* = .837. Our selection of 81 species therefore represents a random sample of Fringillidae phylogenetic diversity.

### 2.3 Construction of syndromes

We selected four sets of variables that together describe a common life history, ecological, or behavioral pattern, which we call syndromes according to Sih, Bell, and Johnson (2004). Each one describes either r‐K‐gradient, ecological specialization, sexual selection, or dispersal/social behavior (Appendix S3). To maximize independence between syndromes, we took care not to include the same variable in the calculation of different syndromes. For each syndrome, all attributed variables were submitted to PCA, with Spearman's coefficient as a measure of correlation, to extract the correlated variation between dominant variables, that is, the functions of the principal components. Ideally, only the first principal component would have a distinctively high eigenvalue (Sih et al., 2004) and could be used as single proxy for ecological specialization, r‐K gradient, sexual selection, and dispersal/social behavior, respectively. For each synthetic variable, species were split into two groups by k‐means clustering (Steinhaus, 1956), maximizing the ratio of between‐group versus within‐group variance. These two groups were considered as two levels of each syndrome, which could then be treated as a discrete variable. Calculations were performed using XLSTAT^®^ for Excel^®^ (Addinsoft^®^, Paris).

### 2.4 Reconstruction of the ancestral states of syndromes

Reconstruction of ancestral states (H1) and test for correlated evolution of the four syndromes (H2) were performed using BayesTraits 2.0 (Pagel & Meade, 2006; Pagel, Meade, & Barker, 2004). Specifically, for each syndrome ancestral states and transition rates between states were estimated by maximum likelihood (H1). To test for correlated evolution of the four syndromes (H2), for each pair of syndrome *i* and *j* we computed the transition rates between states of syndrome *i* under 1) a model where transitions in syndrome *i* were independent from transitions in syndrome *j* (independent model) and 2) a model where transitions in syndrome *i* depended on transitions in syndrome *j* (dependent model, Pagel & Meade, 2006). We then selected the best model by performing a likelihood ratio test (LRT). If traits evolve under a dependent model, this means that their evolution is correlated, in a way depicted by the estimated transition rates (see section 3).

### 2.5 Phylogenetic principal components analysis (pPCA)

The four syndromes (the first principal components of the four original PCAs) were used as continuous variables in a pPCA (Jombart, Pavoine, Devillard, & Pontier, 2010), allowing to discern a phylogenetic signal common to all four syndromes. Phylogenetic signal (autocorrelation) was tested on each synthetic variable by Abouheif's test (Abouheif, 1999; Pavoine, Ollier, Pontier, & Chessel, 2008). These methods were performed using the adephylo package of R (R Core Team, 2016). The reconstruction of ancestral states of the four syndromes suggested that strategy B should be subdivided in two groups. Thereby the first three components of pPCA were used to split the species in three strategies A, B1, and B2 by k‐means clustering.

### 2.6 Association of syndromes with tropical affinity

The tropical affinity of species was determined according to geographical records (Appendix S2). Species were considered as having a tropical affinity when more than half of their distribution area was located in the tropics. To test the association of each syndrome with tropical affinity while correcting for phylogenetic autocorrelation, a phylogenetic ANOVA was performed on the variables describing the four syndromes (first principal component of PCA, see section 3), using tropical affinity as a fixed, two‐level factor, with the function aov.phylo implemented in the R package geiger (Harmon, Weir, Brock, Glor, & Challenger, 2008).

## 3 RESULTS

### 3.1 Phylogeny and representativeness of species diversity

As expected, the topology of the time‐calibrated phylogeny is congruent with that obtained by Zuccon et al. (2012). Also the topology of the tree generated using the supermatrix, which includes 169 of 204 (82%) species, is largely congruent and recovers the same major clades. Minor differences involve a few nodes with low or no support and the branching order of a few clades. The comparison of the time‐calibrated and supermatrix‐based trees confirms that the 81 species in the time‐calibrated tree have been sampled across the entire family, that all major lineages are represented in the reduced dataset and that the species retained in the subsequent analysis are a fair representation of the family diversity and disparity. This result is a logical consequence of the taxa choice operated by Zuccon et al. (2012), which was driven by taxonomic incentive without taking into account ecological or life history traits.

### 3.2 Construction of proxies for syndromes

#### 3.2.1 Ecological specialization

**Figure 1:**
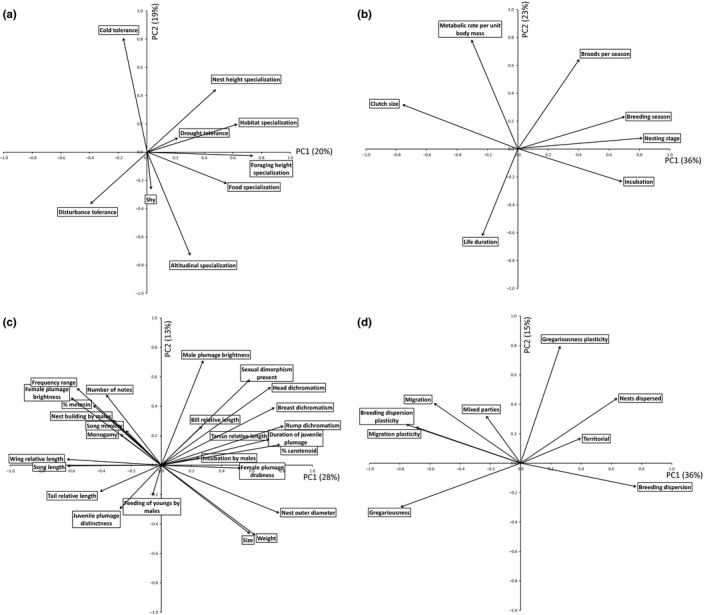
Projection in the plane of the first two principal components of four PCAs of the trait variables describing (a) ecological specialization, (b) r‐K gradient, (c) sexual selection, (d) social/dispersal behavior

The PCA bi‐plot for ecological specialization shows that all indicators of specialization (“foraging height specialization,” “food specialization,” “habitat specialization,” “nest height specialization,” “altitudinal specialization”) are positively correlated with the first principal component PC1 (explaining 20% of the total variance) while indicators of generalism (tolerance), to the exception of “drought tolerance,” are negatively correlated with it (Figure 1a, Table 1). The second principal component (PC2, explaining 19% of the total variance) opposes “cold tolerance” to “altitudinal specialization” and can be interpreted as a specific index of altitudinal specialization, from species restricted to lowlands (lowland specialists) to species with a large altitudinal range (altitudinal generalists). We selected PC1 as the synthetic variable summarizing best the information given by all traits describing ecological specialization (called PC1spec hereafter).

**Table 1:** List of variables selected for the description of four syndromes, with their loadings (= Spearman's rank correlation coefficients) along the first PCA component (one separate analysis per syndrome)

Ecological specialization syndrome	PC1spec	r‐K gradient syndrome	PC1rK
Disturbance tolerance	−0.39	Clutch size	−0.76
Cold tolerance	−0.17	Metabolic rate per unit body mass	−0.31
Shy	0.03	Life duration	−0.24
Drought tolerance	0.22	Broods per season	0.41
Altitudinal specialization	0.30	Incubation	0.68
Nest height specialization	0.48	Breeding season	0.70
Food specialization	0.55	Nesting stage	0.81
Habitat specialization	0.63		
Foraging height specialization	0.74		

Sexual dimorphism syndrome	PC1sex	Dispersal/social behavior syndrome	PC1beh
Wing relative length	−0.63	Gregariousness	−0.80
Song length	−0.62	Breeding dispersion plasticity	−0.75
Female plumage brightness	−0.60	Migration plasticity	−0.69
Frequency range	−0.56	Migration	−0.57
% melanin	−0.45	Mixed parties	−0.23
Tail relative length	−0.41	Gregariousness plasticity	0.26
Number of notes	−0.36	Territorial	0.40
Nest building by males	−0.32	Nests dispersed	0.64
Juvenile plumage distinctness	−0.27	Breeding dispersion	0.76
Monogamy	−0.27		
Song mimicry	−0.23		
Feeding of youngs by males	−0.06		
Incubation by males	0.26		
Bill relative length	0.27		
Male plumage brightness	0.28		
Tarsus relative length	0.32		
Female plumage drabness	0.53		
Weight	0.58		
Sexual dimorphism present	0.59		
Size	0.62		
Duration of juvenile plumage	0.71		
Head dichromatism	0.72		
Breast dichromatism	0.75		
Nest outer diameter	0.78		
% carotenoid	0.79		
Rump dichromatism	0.81		

#### 3.2.2 r‐K gradient

The PC1‐PC2 bi‐plot for the “r‐K‐gradient” syndrome (Figure 1b) shows that “nesting stage” and “clutch size” (see Appendix S2 for definitions) display the highest correlation value with PC1, which explains 36% of the total variation (*r*_s_ ≈ 0.8, Table 1). All indicators of K‐selection (“nesting stage length,” “incubation length,” “number of broods per season”) but one (“life duration”) are positively correlated with PC1, while indicators of r‐selection (“clutch size” and “metabolic rate per unit body mass”) are negatively correlated with it. PC2, which explains 23% of the total variation, is more trait specific, opposing “metabolic rate per unit weight” to “life duration,” displaying the trivial fact that species with high metabolic rate have low life duration. It should be noted that the two variables which are highly correlated with PC2 were poorly documented (for only 38% and 28% of species, respectively, see Appendix S3) compared to the other variables describing the r‐K gradient (68% to 96%). We thus selected PC1 as a synthetic descriptor for the r‐K‐gradient, which will be further called PC1rK.

#### 3.2.3 Sexual selection

In the PC1‐PC2 bi‐plot (Figure 1c), the variables “rump dichromatism,” “% carotenoid,” and “nest outer diameter” exhibit the highest correlation value (*r*_s_ ≈ 0.8) with PC1 (explaining 28% of the total variance). All variables featuring sex dimorphism (including “% carotenoid”) as well as those related to size (“size,” “weight,” “nest diameter”) are positively correlated with PC1 while variables featuring the absence or weakness of sex dimorphism (“female plumage brightness,” “nest building by males”), including “% melanin,” are negatively correlated with it (Table 1), suggesting that PC1 can be used as a proxy for sexual (Fisherian) selection. However, it must be noted that variables measuring the elaboration of male song (“song length,” “frequency range,” “number of notes”) are negatively correlated with PC1, suggesting that this component opposes two bodies of sexual‐selected traits, rather than sexual versus natural selection, as previously thought (Ponge, 2013). It must also be noted that among variables featuring the shape of the body, “wing relative length” and “tail relative length” (on the negative side) are opposed to “tarsus relative length” and “bill relative length” (on the positive side) along this axis, suggesting that the development of appendages used for flight (wing, tail) is opposed to those used for other mechanical functions (bill, tarsus) along this gradient of sexual dimorphism.

The significance of this complex factor, which we will provisionally refer to “sexual dimorphism” rather than to “sexual selection,” will be developed in section 4.

The second principal component, explaining only 13% of the total variation, opposes “size” and “weight” to variables describing sexual dimorphism (all with positive scores along PC1), indicating that among sexually dimorphic species smaller species exhibit a lowest level of sexual differentiation. This subsidiary PC2 component represented a weak proportion of trait variation linked to sexual selection and is not used in the following analyses.

#### 3.2.4 Dispersal/social behavior

PC1 explains 36% of the total variation (Figure 1d). This first principal component is highly correlated with “breeding dispersion” on the positive side and “gregariousness” and “breeding dispersion plasticity” on the negative side (*r*_s_ ≈ ±0.8, Table 1). Indicators of territoriality (“territorial,” “nests dispersed,” “breeding dispersion”) exhibit positive scores, in opposition to “migration,” “gregariousness” and two indicators of plasticity on the negative side. PC1 represents a behavioral gradient from resident/territorial/solitary species to dispersive/poorly territorial/gregarious species. PC2 (explaining only 15% of total variation) was trait specific, opposing “gregarious plasticity” to “gregariousness.” PC1 was selected as the synthetic variable best describing the dispersal/social behavioral syndrome, hereafter called PC1beh.

### 3.3 Ancestrality and evolution of syndromes (H1)

**Figure 2:**
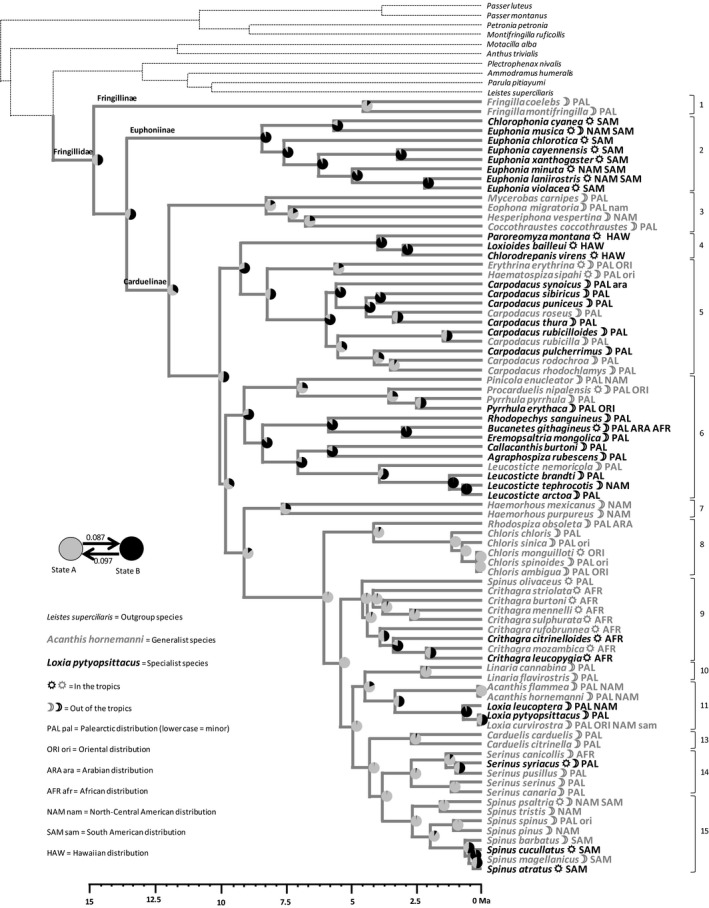
Reconstruction by maximum‐likelihood inference of the ancestral state of the ecological specialization syndrome, using the distribution of species in two groups (see Fig. S1a). Generalism (state A) is in gray while specialism (state B) is in black. Transition rates from one group to the other at the base of the phylogenetic tree are indicated with arrows of proportional thickness. At each node of the phylogenetic tree, a pie chart indicates the confidence given to specialism or generalism as the ancestral state. Transition rates Clade numbers according to Zuccon et al. (2012) are indicated in a column on the right side of the figure. A molecular clock is represented at the bottom of the figure. Geographic distribution and tropical affinity are indicated for each species (see included legend for the significance of codes)

PC1spec was used to split the 81 species into 33 “specialists” and 48 “generalists” (Fig. S1). The transition rate from generalism toward specialism is 0.087, and 0.097 in the reverse direction. The ancestral state of ecological specialization is therefore uncertain (Figure 2). However, the most likely ancestral state for clade 1 (Fringillinae), the most basal clade, and for the core group (Carduelinae) is generalism, with a probability of 0.82 and 0.66, respectively, as shown by pie charts (Figure 2). Uncertainty at the root of the phylogenetic tree is mainly due to clade 2 (Euphoninae) which is currently composed of specialists. Within the core group (Carduelinae) specialism is nearly always in a derived position, and the only cases of reversal being within clade 5, while most extant species in clades 3, 8, 9, 10, 13, 14, and 15 are generalists.

**Figure 3:**
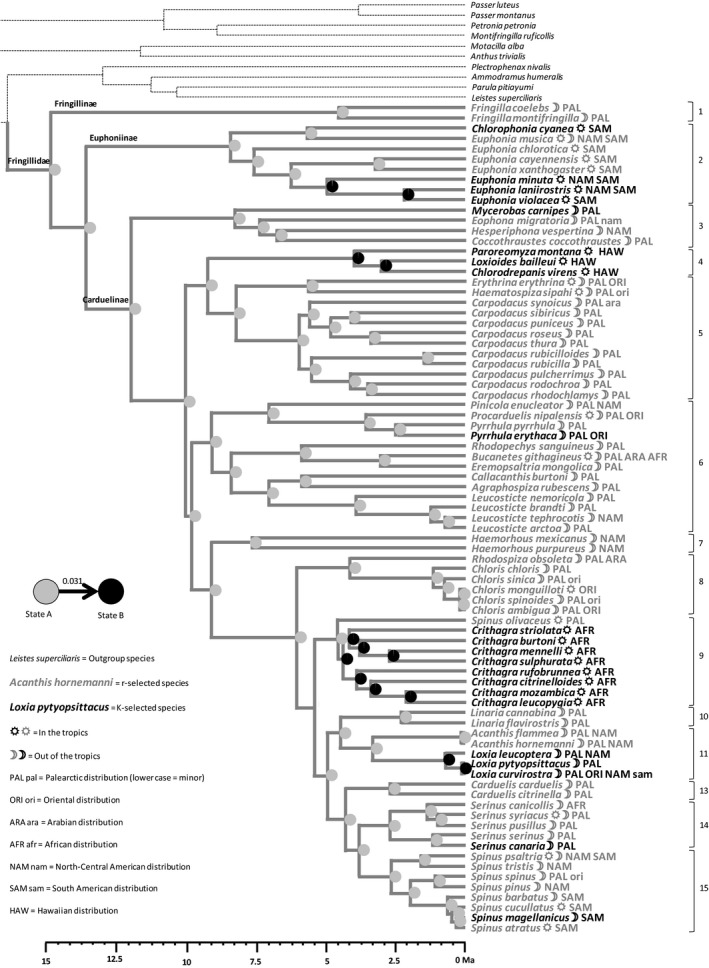
Reconstruction by maximum‐likelihood inference of the ancestral state of the r‐K‐gradient syndrome, using the distribution of species in two groups (see Fig. S1b). r‐selection (state A) is in gray while K‐selection (state B) is in black. At each node of the phylogenetic tree, a pie chart indicates the confidence given to K‐selection or r‐selection as the ancestral state. Otherwise as for Figure 2

PC1rK was used to split the 81 species into 22 “K‐selected” and 59 “r‐selected” species (Fig. S1). Maximum‐likelihood reconstruction showed that r‐selection is the most likely ancestral state for the whole group and for most clades, with a transition rate of 0.031 from r‐selection toward K‐selection and nil in the reverse direction (Figure 3). K‐selection appears as a derived state within Euphoninae tanagers (clade 2), Hawaiian honeycreepers (clade 4), African serins (clade 9), and crossbills (clade 11).

**Figure 4:**
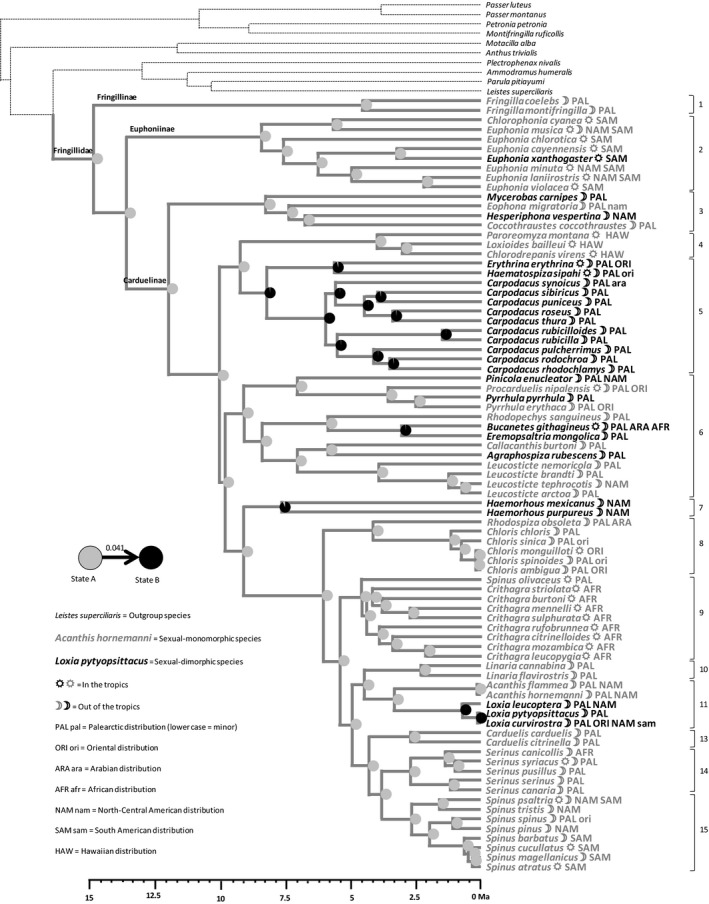
Reconstruction by maximum‐likelihood inference of the ancestral state of the sexual dimorphism syndrome, using the distribution of species in two groups (see Fig. S1c). Sexual monomorphism (state A) is in gray while sexual dimorphism (state B) is in black. At each node of the phylogenetic tree, a pie chart indicates the confidence given to sexual monomorphism or dimorphism as the ancestral state. Otherwise as for Figure 2

PC1sex was used to split the 81 species into 25 “sexually dimorphic” and 56 “sexually monomorphic” species (Fig. S1). Sexual monomorphism is the most likely ancestral state for the whole group and for most clades, with a transition rate of 0.041 from monomorphism toward dimorphism and nil in the reverse direction (Figure 4). Sexual dimorphism appears as a derived state limited to rosefinches (clades 5 and 7), crossbills (clade 11) and isolated species within some other clades.

**Figure 5:**
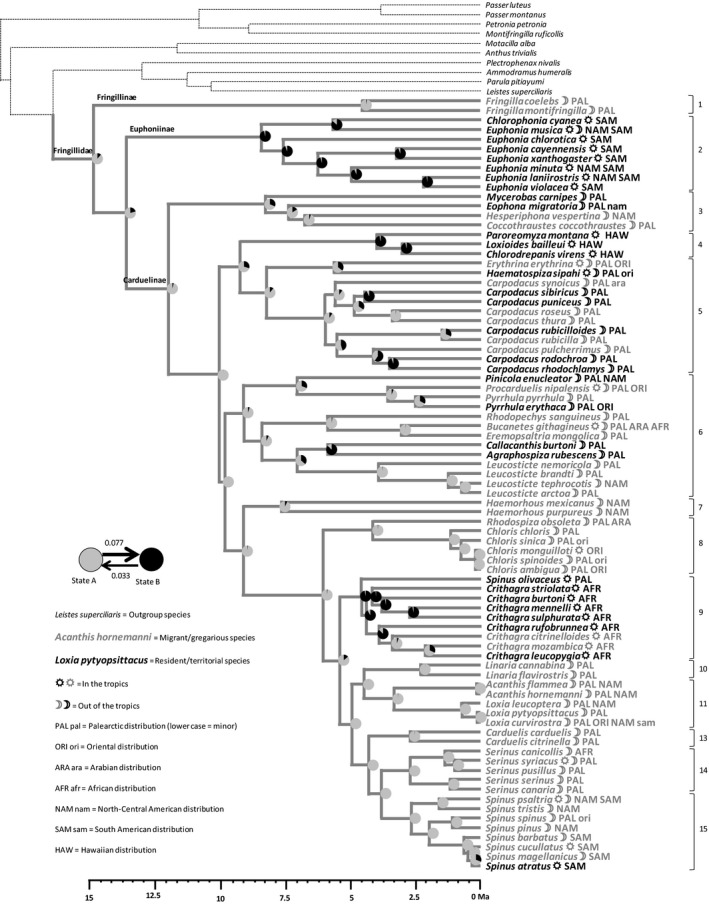
Reconstruction by maximum‐likelihood inference of the ancestral state of the dispersal/social behavioral syndrome, using the distribution of species in two groups (see Fig. S1d). Dispersiveness/gregariousness (state A) is in gray while residence/territoriality (state B) is in black. At each node of the phylogenetic tree, a pie chart indicates the confidence given to residence/territoriality or dispersiveness/gregariousness as the ancestral state. Otherwise as for Figure 2

PC1beh was used to split the 81 species into 31 “resident/territorial” and 50 “dispersive/gregarious” species (Fig. S1). Dispersiveness/gregariousness is the most likely ancestral behavioral state, with a transition rate of 0.077 from dispersiveness/gregariousness toward residence/territoriality and only 0.033 in the reverse direction (Figure 5). Similarly to K‐selection, residence/territoriality appears as a derived state in Euphoninae tanagers (clade 2), Hawaiian honeycreepers (clade 4), and African serins (clade 9), but not in crossbills (clade 11). Derivation toward residence/territoriality is also apparent within rosefinches belonging to clade 5.

### 3.4 Correlated evolution of syndromes (H2)

#### 3.4.1 Modeling and graphical study of the correlated evolution of syndromes

We modeled correlated transitions between states (ancestral vs. derived) in couples of syndromes (Table 2). Several syndromes tend to follow common paths, in particular (1) sexual dimorphism and ecological specialization, (2) r‐K‐gradient and dispersal/social behavior, and (3) ecological specialization and dispersal/social behavior. Reversal from derived to ancestral state never or hardly occurs when one member of these three couples of syndromes is in derived state (see transition rates in Table 2).

**Table 2:** Modeling correlations between transitions from a state (ancestral A or derived B) to the opposite state for four syndromes (ecological specialization, r‐K gradient, sexual dimorphism, dispersal/social behavior). Likelihood ratio test (LRT) was used to test for significance. Significance level for LRT *p*‐value was fixed to .05. Bold types indicate significant relationships between transition states of syndromes. Details are then given on transition rates for pairs of syndromes where transitions between character states are significantly correlated

Syndrome 1	Syndrome 2	Likelihood independent	Likelihood dependent	LRT statistic	LRT *p*‐value
Sexual dimorphism	r‐K gradient	−64.798251	−62.977532	3.641438	0.456700628
Sexual dimorphism	Dispersal/social behavior	−73.281643	−71.241161	4.080964	0.395159442
**Sexual dimorphism**	**Ecological specialization**	**−82.588379**	**−77.793806**	**9.589146**	**0.047947376**
**r‐K gradient**	**Dispersal/social behavior**	**−79.528616**	**−72.309863**	**14.437506**	**0.006022008**
r‐K gradient	Ecological specialization	−88.835352	−84.850098	7.970508	0.092664518
**Dispersal/social behavior**	**Ecological specialization**	**−97.318748**	**−83.446792**	**27.743912**	**1.40558E‐05**

Conditional syndrome state	Dependent syndrome transition	Dependent transition rate			
Sexual selection and ecological specialization					
Sexual dimorphism = A	Ecological specialization A → B	0.068022			
Sexual dimorphism = A	Ecological specialization B → A	0.049974			
Sexual dimorphism = B	Ecological specialization A → B	1.616921			
Sexual dimorphism = B	Ecological specialization B → A	1.472791			
Ecological specialization = A	Sexual dimorphism A → B	0.015748			
Ecological specialization = A	Sexual dimorphism B → A	0^a^			
Ecological specialization = B	Sexual dimorphism A → B	0.060825			
Ecological specialization = B	Sexual dimorphism B → A	0.000164^b^			
r‐K gradient and dispersal/social behavior					
r‐K gradient = A	Dispersal/social behavior A → B	0.052294				
r‐K gradient = A	Dispersal/social behavior B → A	0.169471				
r‐K gradient = B	Dispersal/social behavior A → B	0.47751				
r‐K gradient = B	Dispersal/social behavior B → A	0^c^				
Dispersal/social behavior = A	r‐K gradient A → B	0.044915				
Dispersal/social behavior = A	r‐K gradient B → A	0^d^				
Dispersal/social behavior = B	r‐K gradient A → B	0^e^				
Dispersal/social behavior = B	r‐K gradient B → A	0.072745				
Ecological specialization and dispersal/social behavior						
Ecological specialization = A	Dispersal/social behavior A → D	0.046407				
Ecological specialization = A	Dispersal/social behavior D → A	0.083369				
Ecological specialization = D	Dispersal/social behavior A → D	0.142005				
Ecological specialization = D	Dispersal/social behavior D → A	0^f^				
Dispersal/social behavior = A	Ecological specialization A → D	3.81304				
Dispersal/social behavior = A	Ecological specialization D → A	9.535607^g^				
Dispersal/social behavior = D	Ecological specialization A → D	0^h^				
Dispersal/social behavior = D	Ecological specialization D → A	0^h^				

a When ecological specialization = A, then sexual dimorphism never shifts from D to A.

b When ecological specialization = D, then sexual dimorphism hardly shifts from D to A.

c When r‐K gradient = D, then dispersal/social behavior never shifts from D to A.

d When dispersal/social behavior = A, then r‐K gradient never shifts from D to A.

e When dispersal/social behavior = D, then r‐K gradient never shifts from A to D.

f When ecological specialization = D, then dispersal/social behavior never shifts from D to A.

g When dispersal/social behavior = A, then ecological specialization often shifts from D to A.

h When dispersal/social behavior = D, then ecological specialization neither shifts from A to D nor from D to A.

**Figure 6:**
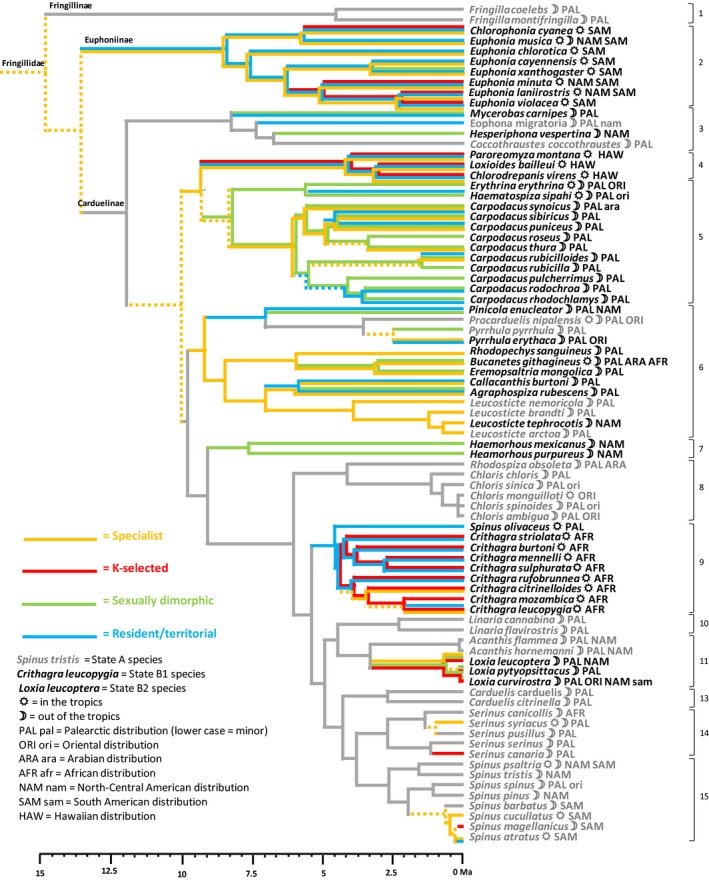
Common representation of paths followed by ecological specialization, r‐K‐gradient, sexual dimorphism, and dispersal/social behavior along the phylogenetic tree of 81 fringillid species. Color lines are for derived states, gray lines for ancestral states, they were dashed in case of incertitude. Species names are in gray (A strategy), black italics (B1 strategy), or black roman (B2 strategy). Otherwise as for Figure 2

The correlated evolution of the four syndromes was then visually addressed by reporting on the same graph in the form of colored trajectories (Figure 6) the maximum‐likelihood patterns displayed by Figures 2, 3, 4, 5. To the exception of generalism, for which uncertainty remains at the base of the tree, the ancestrality of r‐selection, sexual monomorphism, and dispersiveness/gregariousness is more likely than the respective alternative state of each syndrome. Although the correlated evolution of more than two syndromes could not be modeled by our method, Figure 6 shows that three syndromes can follow the same path, although all the four syndromes never follow a common path. Hence, our former, simplistic hypothesis of the existence of two opposite strategies, evolving from an ancestral strategy A (generalists, r‐selected, sexually monomorphic, migratory/gregarious) to a derived strategy B (specialists, K‐selected, sexually dimorphic, resident/territorial) should be refined by considering that these four suites of traits diverged in the course of evolution, generating more than one derived strategy.

#### 3.4.2 Multivariate analysis of phylogenetic signaling

A phylogenetic PCA (pPCA) performed on the four syndromes (PC1spec, PC1rK, PC1sex, PC1beh) was used for a finer delineation of groups of species that are associated to syndromes having evolved correlatively. The four syndromes exhibit each a significant phylogenetic signal (Abouheif's test, *p* < .01). The first three principal components of pPCA exhibit positive eigenvalues (Table 3, Figure 8c), indicating the existence of a phylogenetic signal on each of them. The first component of pPCA displays a phylogenetic signal common to all four syndromes (Figure 8a,b), while second (Figure 8a) and third components (Figure 8b) partial out phylogenetic signals of some groups of syndromes.

**Table 3:** Phylogenetic principal components analysis. Scores of the four syndromes along the first three principal components. Eigen values are given in the last row

	PC1	PC2	PC3
Ecological specialization	−0.4976956	−0.03918335	0.26095389
r‐K gradient	−0.22146655	−0.44248631	0.77233494
Sexual dimorphism	−0.80095985	−0.12831694	−0.47739305
Dispersal/social behavior	−0.248425	0.88668218	0.3278684
Eigen value	0.1587	0.0789	0.0642

The first principal component pPC1 opposes A‐strategists (generalist, r‐selected, sexually monomorphic, gregarious/migratory species), to B‐strategists (species with opposite trait modalities), as expected from our Hypothesis H2 (Figure 8a,b). However, the two following principal components pPC2 and pPC3 show that strategy B could be divided into two strategies, which we call B1 and B2 (Figure 8a,b). Strategy B1 is characterized by a combination of sexual dimorphism and r‐selection (Figure 8b) while strategy B2 is characterized by the common occurrence of residence/territoriality (Figure 8a) and K‐selection (Figure 8b). The originality of strategy B1 is that it combines trait modalities of strategy A (r‐selection) and strategy B (sexual dimorphism), while strategy B2 is characterized by trait modalities of strategy B, to the exception of sexual dimorphism.

**Figure 7:**
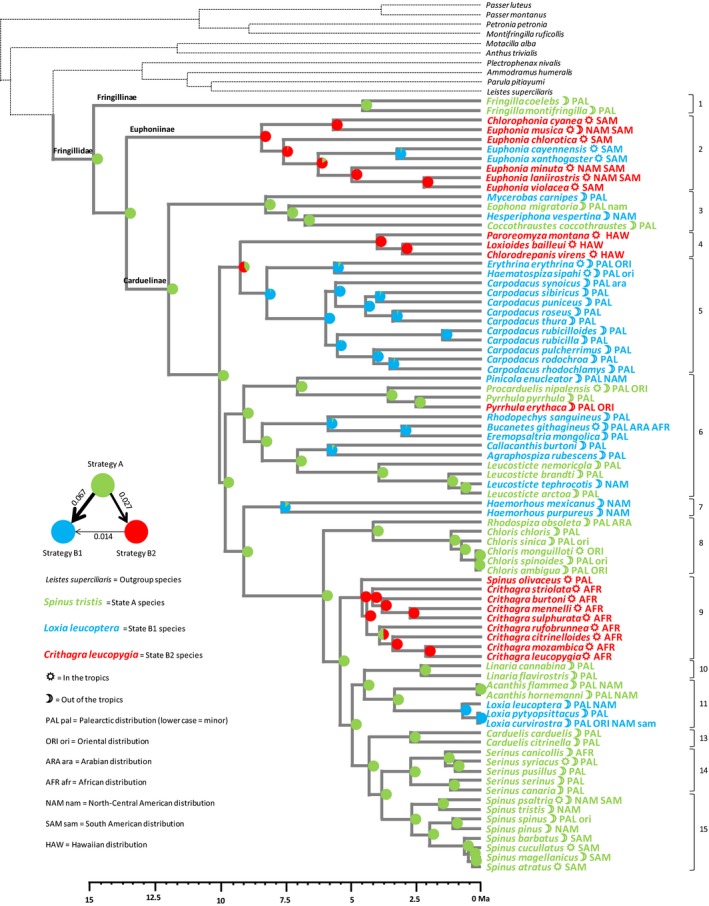
Reconstruction by maximum‐likelihood inference of the ancestral state of the three strategies A (green), B1 (blue), and B2 (red), using the distribution of species in three groups according to k‐means clustering based on the first three principal components of phylogenetic PCA. At each node of the phylogenetic tree, a pie chart indicates the confidence given to residence/territoriality or dispersiveness/gregariousness as the ancestral state. Otherwise as for Figure 2

We used the 3D space of the first three principal components of pPCA to split the whole set of species into those three strategies (A, B1, B2) with the k‐means method of variance partition (with k = 3). The three strategies are indicated on Figure 6. The reconstruction of ancestral traits gives a full support to the ancestrality of strategy A and to derived positions for strategies B1 and B2 (Figure 7). The highest transition rate is from A toward B1 (0.067), followed by A toward B2 (0.027), while reversal rates are nil. Transition from B2 toward B1 is possible but less frequent than transition from A to either strategy B (transition rate 0.014), while transition from B1 toward B2 never occurs. Strategy A is expressed in clades 1, 8, 10, 13–15, partly in clades 3 and 11. Strategy B1is expressed in clades 5 and 7, partly in clades 6 and 11, while the B2 strategy is expressed in clades 2, 4, and 9.

### 3.5 Association of syndromes with tropical affinity (H3)

We tested whether there was a latitudinal signal (tropical affinity) in the distribution of syndrome states by performing phylogenetic ANOVAs. K‐selection and residence/territoriality exhibit the same significant affinity for tropical areas, while sexual dimorphism exhibits a higher affinity for nontropical areas, and specialism does not display any significant latitudinal signal (Table 4).

**Table 4:** Relationship between syndrome groups and tropical affinity, tested by phylogenetic ANOVA (ANOVA corrected for phylogenetic autocorrelation)

	Tropical affinity	*F*‐value	Probability
Generalist vs. specialist	0.41 vs. 0.53	0.17	0.81 NS
r‐Selected vs. K‐selected	0.35 vs. 0.77	13.25	0.025*
Sexual monomorphism vs. sexual dimorphism	0.56 vs. 0.23	12.52	0.026*
Dispersive/gregarious vs. resident/territorial	0.33 vs. 0.68	12.02	0.032*

NS = not significant at 0.05 level; *p < .05.

## 4 DISCUSSION

### 4.1 Ancestral syndromes in Fringillidae

Results support our H1 hypothesis: r‐selection and dispersiveness/gregariousness are ancestral and tend to shift toward derived attributes in the course of evolution. Even though most species/traits are spread along a continuous r‐K gradient (Jones, 1976), it has been theoretically (Blanck, Tedesco, & Lamouroux, 2007) and empirically demonstrated (Flegr, 1997) that species whose reproductive strategy lies in the middle of the r‐K continuum are at disadvantage, justifying the distinction of two opposite strategies. Our qualitative approach shows two groups of r‐selected and K‐selected species. Comparisons between tropical and nontropical bird species showed an association of K‐strategy (approximated by basal metabolic rate) with climatically stable environments (Wiersma, Muñoz‐Garcia, Walker, & Williams, 2007), supporting theoretical expectations (Southwood, May, Hassell, & Conway, 1974).

The reconstruction of ancestral traits for the behavioral syndrome shows that behavioral plasticity and gregariousness have a basal position while rather standardized and solitary behaviors are in derived position (Figure 5), as shown by Simpson, Johnson, and Murphy (2015) in Parulidae. Here too, this syndrome is clearly related to the stability of the environment, gregariousness and dispersal ability being advantageous for motile organisms facing environmental hazards (Stevens et al., 2014). We may add that once bursts of speciation appear in novel environments, ancestral traits associated with migration (in particular orientation and memorization of geographic features, together with collective behavior) may disappear in favor of sophisticated signaling traits associated with territoriality if the colonized environment turns out to be more stable than the original environment, a case of relaxed selection pressure (Wiersma, Nowak, & Williams, 2012).

The ancestral reconstruction of the “sexual dimorphism” syndrome shows that a group of sexually selected traits related to male song elaboration (Table 1) is in ancestral position while a group of sexually selected traits related to plumage color and body size is in derived position (Figure 4). This is not in agreement with our original hypothesis of natural selection opposed to sexual selection, the former process being a response to fluctuations of the environment while the latter would drive directional evolution in stable environments (Møller & Garamszegi, 2012). However, it has been shown that sexual selection is also an adaptive response to environmental constraints and fluctuations (Badyaev & Ghalambor, 1998), and that synergistic combinations of natural and sexual selection are widespread (Botero & Rubenstein, 2012). The opposition between visual (size, color) and acoustic signals (song display) has been already observed by Badyaev et al. (2002) in Carduelinae. Such a reversal from one type of sexually selected signal to another is known as the “transfer hypothesis” (Shutler & Weatherhead, 1990). The observed contrast between song elaboration and color display is paralleled by a contrast between melanin and carotene pigments in plumage coloration (Table 1). Both melanin‐ and carotene‐based plumage colors have been shown to predict success in dominance interactions between males and influence favorably female choices (Hill, 1990; Tarof, Dunn, & Whittingham, 2005) and are honest signals of male good condition and resistance to parasites (Roulin, 2015; Safran, McGraw, Wilkins, Hubbard, & Marling, 2010). However, carotene must be found in the environment while melanin is produced by birds themselves (Griffith, Parker, & Olson, 2006; Roulin, 2015). We hypothesize that in environments where food resources are scarce, the ability of males to find high‐quality resources and allocate this quality to offspring is advantageous to survival and is favored by mates (carotene‐based sexual selection). Conversely, in environments where resources are abundant, at least in breeding areas, melanin, which is not context dependent but pleiotropically linked to body condition and strongly heritable (Roulin, 2015), is favored by mates (melanin‐based sexual selection).

The ancestrality of dispersiveness (see above) suggests that ancestral true finches (Fringillidae) were monomorphic (melanin‐based conspicuous plumage in both sexes) had elaborated song and were well‐equipped for flying to remote breeding areas where resources are seasonally abundant, while dimorphic (carotene‐based colored) resident birds are better equipped for foraging in areas where resources are scarcely distributed. The ancestrality of monochromatism (males and females both harboring a bright coloration) has been demonstrated in other bird groups (Friedman, Hofmann, Kondo, & Omland, 2009; Simpson et al., 2015).

Our hypothesis of generalism as ancestral in the Fringillidae cannot be rejected, because of two arguments. First, generalism is an ancestral state in Carduelinae, the core group and, second, the two species of the most basal subfamily, the Fringillinae, which are included in our study (*F. coelebs* and *F. montifringilla*), are clearly generalists. It should also be noted that reversal from specialism to generalism is observed in clade 5 only (Figure 2). Therefore, despite uncertainty of ancestral reconstruction, specialism is a derived state in all clades but clade 5. An important point is that generalism is still the commonest state in the crown group (clades 7 to 15, Figure 2), that is, in clades most remote from the common ancestor and thus resulting from a high number of speciation events.

The ancestrality of generalism has been demonstrated in a variety of monophyletic groups, among plants (Schneeweiss, 2007; Tripp & Manos, 2008) and animals (Hwang & Weirauch, 2012; Kelley & Farrell, 1998; Loiseau et al., 2012; Prinzing, D'Haese, Pavoine, & Ponge, 2014; Yotoko & Elisei, 2006), birds included (Brumfield et al., 2007; Jønsson, Fabre, Ricklefs, & Fjeldså, 2011), while fewer studies conclude to the ancestrality of specialism (Sedivy, Praz, Müller, Widmer, & Dorn, 2008; Stireman, 2005). Despite repeated assessment of phylogenetic conservatism of niche requirements (Brumfield et al., 2007; Peterson, Soberón, & Sánchez‐Cordero, 1999; Prinzing, Durka, Klotz, & Brandl, 2001; Prinzing et al., 2014; Wiens & Graham, 2005), confirmed in the present study, it has even been shown that habitat specialization is a labile ecological trait that may change in the short‐term within the same species (Barnagaud, Devictor, Jiguet, & Archaux, 2011). This might explain why ancestral attributes of ecological specialization are less clear‐cut than for r‐K, sexual selection, and behavioral syndromes.

### 4.2 The correlated evolution of syndromes in Fringillidae

**Figure 8:**
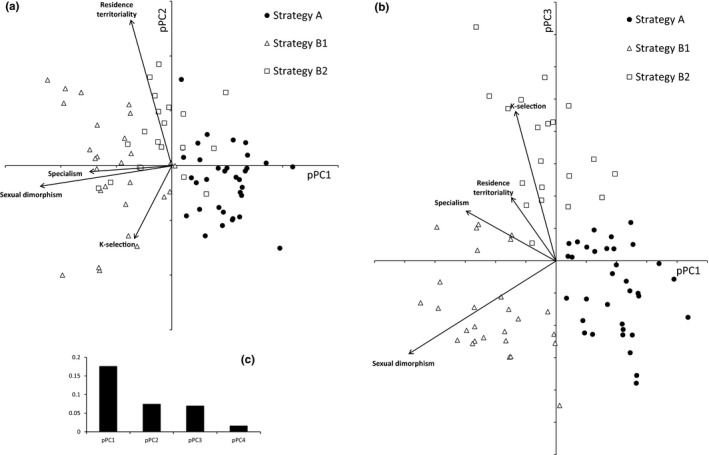
Projection in the plane of the first and second (a), and first and third, (b) principal components of phylogenetic PCA of the synthetic variables (see text for details) describing the four studied syndromes. Eigenvalues are diagrammatically represented (c)

Even though we do not record a correlated evolution of all four syndromes, a common phylogenetic signal is detected (Table 3) and three syndromes exhibit a correlated evolution along the phylogenetic tree (r‐K‐gradient, ecological specialization, and dispersal/social behavior, Table 3, Figure 6). Sexual dimorphism appears to have evolved in association with ancestral trait modalities of the three other syndromes (Figures 6 and 8) and is not associated with K‐selection and residence/territoriality (with the exception of the genus *Loxia* within clade 11, associated with K‐selection, see Figure 3). This feature explains why a common evolution of the four syndromes was not detected. However, r‐selected traits, sexual monomorphism, and dispersiveness/gregariousness are all present in ancient lineages in the phylogenetic tree, supporting at least partially our hypothesis H2 that syndromes evolved correlatively along the phylogenetic tree of Fringillidae.

Our results point to the existence of a common ancestor to Fringillidae with all features of the A strategy (generalism, r‐selection, monochromatism with elaborated song, dispersiveness/gregariousness), with a further evolution of a group of clades where sexual dimorphism is prominent while other features of the A strategy are kept (strategy B1), and another group where most features of the B strategy evolved to the exception of sexual dimorphism (strategy B2; Figure 7).

### 4.3 Geographical distribution and past history of the Fringillidae

We may wonder whether the observed discrepancy between a lineage with sexually selected dimorphism (B1) and another with K‐selected, specialist, and resident/territorial traits (B2) could be explained by geographical affinities of the species, and the way different environments were colonized in the course of the evolution of this now worldwide bird family. If we examine the relationship between tropical affinity and the balance between ancestral and derived states of our four syndromes (Table 4), it appears that most tropical species are K‐selected and resident territorial while most nontropical species are dimorphic sexually, and ecological specialization is undifferentiated. This result supports only partly our third hypothesis (H3) that derived attributes are associated with tropical affinity, suggesting that the B2 type lives (and probably evolved) mainly in tropical areas, while the B1 branch is located (and probably evolved) mainly out of the tropics.

The existence of a sexually dimorphic branch living out of the tropics is supported by studies on other bird families (Bailey, 1978; Friedman et al., 2009), while Price, Lanyon, and Omland (2009) pointed on singing by both sexes as the tropical ancestral state in New World blackbirds. Species we classified in the sexually dimorphic group (Fig. S1c) are mostly adapted to life in harsh environments. An extraordinary variety of true rosefinches (clade 5) are living in the Himalayas, which are thought to be a source of species radiation (Tietze et al., 2013). Within clade 6, the trumpeter finch *Bucanetes githagineus* and the Mongolian finch *Eremopsaltria mongolica* live in deserts or semi‐deserts, and the Blanford's rosefinch *Agraphospiza rubescens* lives in the Himalayas. Other species of this group have a circumpolar distribution and live in coniferous forests (the white‐winged crossbill *Loxia leucoptera*, the red crossbill *Loxia curvirostra*). However, mountain finches (*Leucosticte*) live in Asian tundras and high mountains and display no or only weak sexual dimorphism, thus tolerance of harsh habitats does not necessarily entail sexual dimorphism. Contrary to erroneous ideas resulting from the ordinary confusion between stress and disturbance (Borics, Várbiró, & Padisák, 2013), such harsh habitats are highly predictable (Greenslade, 1983), allowing seasonal short‐distance (altitudinal) or long‐distance (latitudinal) migration to cope with foraging and breeding bird requirements. According to Ponge (2013) cyclic, seasonal processes to which organisms are adapted allows anticipation, a key adaptive trait of strategy B.

The existence of K‐selected, resident/territorial (also specialist?) B2‐strategists living in the tropics is attested by many studies on birds (Jetz, Freckleton, & MvKechnie, 2008; Sol et al., 2010; Wiersma et al., 2007, 2012). We may thus consider that strategy B2 is associated with stable benign environments, better exemplified near the Equator in tropical rainforests.

## 5 CONCLUSIONS

Our results provide strong support to strategy A (r‐selection, sexual monomorphism, migratory/gregarious) as ancestral in the fringillid family. However, our former idea of two opposite strategies previously called “barbarians” (ancestral) and “civilized” (derived) by Ponge (2013), and now called A and B, respectively, must be refined. Strategy B, associated with predictability of the environment, should be subdivided in B1 (sexually dimorphic, r‐selected, migratory/gregarious), associated with harsh habitats (mountain and boreal habitats), mostly out of the tropics, and B2 (sexually monomorphic, K‐selected, resident/territorial), associated with most benign habitats, mostly in lowland tropics. Both habitats of B‐organisms are predictable, although in B1 the high seasonality forces species to migrate seasonally (either altitudinal or latitudinal migration) while in B2 the ancestral migratory behavior has been lost, being unnecessary.

## CONFLICT OF INTEREST

None declared.

## AUTHORS CONTRIBUTION

Jean‐François Ponge performed most part of the redaction, part of the calculations (construction of the synthetic variables as proxies for trait syndromes), made graphical representations of the results, and collected the original data in published literature. Dario Zuccon performed the phylogeny and contributed to the redaction. Marianne Elias performed the reconstruction of ancestral trait syndromes and some other calculations, and contributed to the redaction. Sandrine Pavoine performed the phylogenetic PCA and some other calculations and contributed to the redaction. Pierre‐Yves Henry contributed to the redaction and to fruitful discussions about methods and concepts. Marc Théry and Éric Guilbert contributed to the redaction and to fruitful discussions.
