# Supplementary material for: Ancestrality and evolution of trait syndromes in finches (Fringillidae)
Source: Ecol Evol. 2017 Oct 21;7(23):9935–53. doi: 10.1002/ece3.3420 (PMC5723631; doi:10.1002/ece3.3420)
Supplement: Supplementary file 1 [file ECE3-7-9935-s001.docx]

**Appendix S1.** References used for collecting data on Fringillidae. Numbers in square brackets refer to Appendix S2.

[1] Barrientos, R., Barbosa, A., Valera, F., Moreno, E., 2009a. Breeding parameters of the trumpeter finch at the periphery of its range: a case study with mainland expanding and island populations. Journal of Arid Environments 73, 1177–1180.

[2] Barrientos, R., Valera, F., Barbosa, A., Carrillo, C.M., Moreno, E., 2009b. Plasticity of nest-site selection in the trumpeter finch: a comparison between two different habitats. Acta Oecologica 35, 499-506.

[3] Carrillo, C.M., Moreno, E., Valera, F., Barbosa, A., 2007a. Seed selection by the trumpeter finch, Bucanetes githagineus. What currency does this arid-land species value? Annales Zoologici Fennici 44, 377-386.

[4] Barrientos, R., Barbosa, A., Valera, F., Moreno, E., 2007. Temperature but not rainfall influences timing of breeding in a desert bird, the trumpeter finch (Bucanetes githagineus). Journal of Ornithology 148, 411-416.

[5] Valera, F., Carrillo, C.M., Barbosa, A., Moreno, E., 2003. Low prevalence of haematozoa in Trumpeter finches Bucanetes githagineus from south-eastern Spain: additional support for a restricted distribution of blood parasites in arid lands. Journal of Arid Environments 55, 209-213.

[6] Carrillo, C., Barbosa, A., Valera, F., Barrientos, R., Moreno, E., 2007b. Northward expansion of a desert bird: effects of climate change? Ibis 149, 166-169.

[7] Barrientos, R., Kvist, L., Barbosa, A., Valera, F., López-Iborra, G.M., Moreno, E., 2009. Colonization patterns and genetic structure of peripheral populations of the trumpeter ﬁnch (Bucanetes githagineus) from Northwest Africa, the Canary Islands and the Iberian Peninsula. Journal of Biogeography 36, 210–219.

[8] Carrillo, C.M., Valera, F., Barbosa, A., Moreno, E., 2007c. Thriving in an arid environment: high prevalence of avian lice in low humidity conditions. Écoscience 14, 241-249.

[9] Carrascal, L.M., Cayuela, L., Palomino, D., Seoane, J., 2012. What species-specific traits make a bird a better surrogate of native species richness? A test with insular avifauna. Biological Conservation 152, 204-211.

[10] Danet, P., 2006. Les oiseaux des environs de Ouarzazate au début des années 1980. Go-South Bulletin 3, 18-27.

[11] Carrascal, L.M., Palomino, D. 2005. Preferencias de hábitat, densidad y diversidad de las comunidades de aves en Tenerife (islas Canarias). Animal Biodiversity and Conservation 28, 101-119.

[12] Khoury, F., Al-Shamlih, M., 2006. The impact of intensive agriculture on the bird community of a sand dune desert. Journal of Arid Environments 64, 448-459.

[13] Donald, P.F., Sanderson, F.J., Burfield, I.J., Van Bommel, F.P.J., 2006. Further evidence of continent-wide impacts of agricultural intensification on European farmland birds, 1990–2000. Agriculture, Ecosystems and Environment 116, 189-196.

[14] Barbet‐Massin, M., Thuiller, W., Jiguet, F., 2010. How much do we overestimate future local extinction rates when restricting the range of occurrence data in climate suitability models? Ecography 33, 878-886.

[15] Marten, J.A., Johnson, N.K., 1986. Genetic relationships of North American cardueline finches. Condor 88, 409-420.

[16] Møller, A.P., Garamszegi, I.Z., 2012. Sexual selection, range size and population size. Ornis Hungarica 20, 1–25.

[17] Moreno, J., Soler, J.J., 2011. Sources of distinctness of juvenile plumage in Western Palearctic passerines. Biological Journal of the Linnean Society 102, 440-454.

[18] Moreno-Rueda, G., 2010. European bird species have expanded northwards during 1950-1993 in response to recent climatic warming. In: Ulrich, P.K., Willet, P.K. (Eds.), Trends in ornithology research. Nova Science Publishers, New York, pp. 137-151.

[19] Badyaev, A., 1997a. Avian life history variation along altitudinal gradients: an example with cardueline finches. Oecologia 111, 365-374.

[20] Arnaiz-Villena, A., Moscoso, J., Ruiz-del-Valle, V., Gonzalez, J., Reguera, R., Ferri, A., Wink, M., Serrano-Vela, J.I., 2008. Mitochondrial DNA phylogenetic definition of a group of ‘arid-zone’ Carduelini finches. Open Ornithology Journal 1, 1-7.

[21] Badyaev, A., 1997b. Altitudinal variation in sexual dimorphism: a new pattern and alternative hypotheses. Behavioral Ecology 8, 675-690.

[22] Badyaev, A.V., Hill, G.E., Weckworth, B.V., 2002. Species divergence in sexually selected traits: increase in song elaboration is related to decrease in plumage ornamentation in finches. Evolution 56, 412-419.

[23] Irwin, R.E., 1994. The evolution of plumage dichromatism in the New World blackbirds: social selection on female brightness. American Naturalist 144, 890-907.

[24] Hamilton, W.D., Zuk, M., 1982. Heritable true fitness and bright birds: a role for parasites? Science 218, 384-387.

[25] Zamora, J., Lowy, E., Ruiz-del-Valle, V., Moscoso, J., Serrano-Vela, J.I., Rivero-de-Aguilar, J., Arnaiz-Villena, A., 2006. Rhodopechys obsoleta (desert finch): a pale ancestor of greenfinches (Carduelis spp.) according to molecular phylogeny. Journal of Ornithology 147, 448-456.

[26] Badyaev, A.V., Hill, G.E., 2000. Evolution of sexual dichromatism: contribution of carotenoid- versus melanin-based coloration. Biological Journal of the Linnean Society 69, 153-172.

[27] Martin, T.E., Badyaev, A.V., 1996. Sexual dichromatism in birds: importance of nest predation and nest location for females versus males. Evolution 50, 2454-2460.

[28] Badyaev, A.V., 1997. Covariation between life history and sexually selected traits: an example with cardueline finches. Oikos 80, 128-138.

[29] Kirwan, G.M., Gregory, S.M.S., 2005. A new genus for the Mongolian finch Bucanetes mongolicus (Swinhoe, 1870). Bulletin of the British Ornithologists' Club 125, 68-80.

[30] Arnaiz-Villena, A., Álvarez-Tejado, M., Ruíz-del-Valle, V., García-de-la-Torre, C., Varela, P., Recio, M.J., Ferre, S., Martínez-Laso, J., 1998. Phylogeny and rapid Northern and Southern Hemisphere speciation of goldfinches during the Miocene and Pliocene Epochs. Cellular and Molecular Life Sciences 54, 1031-1041.

[31] Møller, A.P., 1998. Evidence of larger impact of parasites on hosts in the tropics: investment in immune function within and outside the tropics. Oikos 82, 265-270.

[32] Arnaiz-Villena, A., Areces, C., Rey, D., Enríquez-de-Salamanca, M., Alonso-Rubio, J., Ruiz-del-Valle, V., 2012. Three different North American siskin/goldfinch evolutionary radiations (genus Carduelis): pine siskin green morphs and European siskins in America. Open Ornithology Journal 5, 73-81.

[33] Møller, A.P., Sorci, G., Erritzøe, J., 1998. Sexual dimorphism in immune defense. American Naturalist 152, 605-619.

[34] Björklund, M., Merilä, J., 1993. Morphological differentiation in Carduelis finches: adaptive vs. constraint models. Journal of Evolutionary Biology 6, 359-373.

[35] Villegas, M., Garitano-Zavala, À., 2010. Bird community responses to different urban conditions in La Paz, Bolivia. Urban Ecosystems 13, 375–391.

[36] Arnaiz-Villena, A., Ruiz-del-Valle, V., Reche, P., Gomez-Prieto, P., Lowry, E., Zamora, J., Areces, C., Rey, D., Parga, C., Serrano-Vela, J.I., 2010. Songbirds Conserved Sites and Intron Size of MHC Class I molecules reveal a unique evolution in vertebrates. Open Ornithology Journal 3, 156-165.

[37] Vuilleumier, F., 1997. How many bird species inhabit the puna desert of the high Andes of South America? Global Ecology and Biogeography Letters 6, 149-153.

[38] Lloyd, H., Marsden, S.J., 2011. Between-patch bird movements within a High-Andean Polylepis woodland/matrix landscape: implications for habitat restoration. Restoration Ecology 19, 74-82.

[39] Deferrari, G., Camilión, C., Pastur, G.M., Peri, P.L., 2001. Changes in Nothofagus pumilio forest biodiversity during the forest management cycle. II. Birds. Biodiversity and Conservation 10, 2093-2108.

[40] Lencinas, M.V., Pastur, G.M., Medina, M., Busso, C., 2005. Richness and density of birds in timber Nothofagus pumilio forests and their unproductive associated environments. Biodiversity and Conservation 14, 2299-2320.

[41] Simeone, A., Valencia, J. C., Schlatter, R., Lanfranco, D., Ide, S., 1997. Depredación de aves sobre larvas de Rhyacionia buoliana (Schiff.) (Lepidoptera: Tortricidae) en plantaciones jóvenes de Pinus radiata D. Don en el sur de Chile. Bosque 18, 67-75.

[42] Hall, J.R., Woods, R.W., Brooke, M. de L., Hilton, G.M., 2002. Factors affecting the distribution of landbirds on the Falkland Islands. Bird Conservation International 12, 151–167.

[43] González-Gómez, P.L., Estades, C.F., Simonetti, J.A., 2006. Strengthened insectivory in a temperate fragmented forest. Oecologia 148, 137-143.

[44] Cofre, H.L., Böhning-Gaese, K., Marquet, P.A., 2007. Rarity in Chilean forest birds: which ecological and life-history traits matter? Diversity and Distributions 13, 203–212.

[45] Pablo Sabat, P., Ramirez-Otarola, N., Barceló, G., Salinas, J., , Bozinovic, F., 2010. Comparative basal metabolic rate among passerines and the food habit hypothesis. Comparative Biochemistry and Physiology, Part A 157, 35-40.

[46] Archuby, D.I., Marti, L.J., Montalti, D., Soave, G.E., Camperi, A.R., Arambarri, A.M., Darrieu, C.A., 2007. Alimentación del Cabecitanegra Austral (Carduelis barbata) durante el otoño. El Hornero 22, 65-68.

[47] Kelt, D.A., Engilis, A. Jr., Monárdez, J., Walsh, R., Meserve, P.L., Gutiérrez, J.R., 2012. Seasonal and multiannual patterns in avian assemblage structure and composition in northern Chilean thorn-scrub. The Condor 114, 30-43.

[48] Clement, P., 1993. Finches and sparrows. Christopher Helm, London.

[49] IUCN, 2013. IUCN Red List of threatened species, Version 2013.1.

[50] Moorcroft, D., Wilson, J.D., Bradbury, R.B., 2006. Diet of nestling linnets Carduelis cannabina on lowland farmland before and after agricultural intensification. Bird Study 53, 156-162.

[51] Lasiewski, R.C., Dawson, R.D., 1967. A re-examination of the relation between standard metabolic rate and body weight in birds. The Condor 69, 13-23.

[52] Khoury, F., Janaydeh, M., Al-Hmoud, A.R., 2009. Nest placement and nesting success in two finch species colonizing a recently established plantation in an arid region. Journal of Ornithology 150, 29-37.

[53] Newton, I., 1967. The adaptive radiation and feeding ecology of some British finches. Ibis 109, 33-96.

[54] Cramp, S., Perrins, C.M., 1994. Handbook of the birds of Europe, the Middle East and North Africa. VIII. Crows to finches. Oxford University Press, Oxford.

[55] Arnaiz-Villena, A., Guillén, J., Ruiz-del-Valle, V., Lowy, E., Zamora, J., Varela, P., Stefani, D., Allende, L.M., 2001. Phylogeography of crossbills, bullfinches, grosbeaks, and rosefinches. Cellular and Molecular Life Sciences 58, 1159-1166.

[56] Ottaviani, M., 2008. Monographie des Fringilles (Fringillinés-Carduélinés): histoire naturelle et photographies, Volume 1. Prin, Ingré.

[57] Arnaiz-Villena, A., Ruiz-del-Valle, V., Moscoso, J., Serrano-Vella, J.I., Zamora, J., 2007. mtDNA phylogeny of North American Carduelis pinus group. Ardeola 54, 1-14.

[58] Zamora, J., Moscoso, J., Ruiz-del-Valle, V., Lowy, E., Serrano-Vela, J.I., Ira-Cachafeiro, J., Arnaiz-Villena, A., 2006. Conjoint mitochondrial phylogenetic trees for canaries Serinus spp. and goldfinches Carduelis spp. show several specific polytomies. Ardeola 53, 1-17.

[59] Ottaviani, M., 2008. Monographie des Fringilles (Carduélinés): histoire naturelle et photographies, Volume 2. Prin, Ingré.

[60] Ottaviani, M., 2011. Monographie des Fringilles (Carduélinés): histoire naturelle et photographies, Volume 3. Prin, Ingré.

[61] Geffen, E., Yom-Tov, Y., 2000. Are incubation and fledging periods longer in the tropics? Journal of Animal Ecology 69, 59-73.

[62] Wu, H.C., Lin, R.C., Hung, H.Y., Yeh, C.F., Chu, J.H., Yang, X.J., Yao, C.J., Zou, F.S., Yao, C.T., Li, S.H., Lei, F.M., 2011. Molecular and morphological evidences reveal a cryptic species in the vinaceous roseﬁnch Carpodacus vinaceus (Fringillidae; Aves). Zoologica Scripta 40, 468–478."

[63] Tietze, D.T., Päckert, M., Martens, J., Lehmann, H., Sun, Y.H., 2013. Complete phylogeny and historical biogeography of true rosefinches (Aves: Carpodacus). Zoological Journal of the Linnean Society 169, 215-234.

[64] Shiu, H.J., Ding, T.S., Sheu, J.E., Lin, R.S., Koh, C.N., Lee, P.F., 2005. Morphological characters of bird species in Taiwan. Taiwania 50, 80-92.

[65] Dunning, J.B. Jr, 2007. CRC handbook of avian body masses, 2nd edition. CRC Press,

[66] Töpfer, T., Haring, E., Birkhead, T.R., Lopes, R.J., Severinghaus, L. Liu , Martens, J., Päckert, M., 2011. A molecular phylogeny of bullfinches Pyrrhula Brisson, 1760 (Aves: Fringillidae). Molecular Phylogenetics and Evolution 58, 271-282.

[67] Ding, T.S., Liao, H.C., Yuan, H.W., 2008. Breeding bird community composition in different successional vegetation in the montane coniferous forests zone of Taiwan. Forest Ecology and Management 255, 2038-2048.

[68] Koh, C.N., Lee, P.F., Wu, S.H., 2006. Does the distribution of breeding bird species richness in Taiwan follow the mid-domain effect? Taiwania 51, 108-116.

[69] Bonaparte, C.L., Schlegel, H., 1850. Monographie des loxiens. Arnz, Leiden.

[70] Foster, M.S., 1987. Feeding methods and efficiencies of selected frugivorous birds. Condor 89, 566-580.

[71] Janni, O., Boano, G., Pavia, M., Gertosio, G., 2008. Notes on the breeding of birds in Yanachaga-Chemillén National Park, Peru. Cotinga 30, 42-46.

[72] Ruelle, M., 1997. Le verdier d'Europe [Carduelis chloris (Linné)] et ses cousins, les verdiers orientaux. Fédération Ornithologique Wallonne, Horion-Hozémont.

[73] Freeman, BG, Class, A.M., Olaciregui, C.A., Botero-Delgadillo, E., 2012. Breeding biology of the blue-naped chlorophonia (Chlorophonia cyanea) in the Santa Marta Mountains. Ornitología Colombiana 12, 10-16.

[74] Terborgh, J., Robinson, S.K., Parker, T.A.III, Munn, C.A., Pierpont, N., 1990. Structure and organization of an Amazonian forest bird community. Ecological Monographs 60, 213-238.

[75] Tello, J.G., 2003. Frugivores at a fruiting Ficus in south-eastern Peru. Journal of tropical Ecology 19, 717-721.

[76] Ricklefs, R.E., Tsunekage, T., Shea, R.E., 2011. Annual adult survival in several new world passerine birds based on age ratios in museum collections. Journal of Ornithology 152, 481-495.

[77] Kessler-Rios, M.M., Kattan, G.H., 2012. Fruits of Melastomataceae: phenology in Andean forest and role as a food resource for birds. Journal of Tropical Ecology 28, 11-21.

[78] Terborgh, J., Weske, J.S., 1969. Colonization of secondary habitats by Peruvian birds. Ecology 50, 765-782.

[79] Cuervo, A.M., Pulgarín, P.C., Calderón-F., D., Ochoa-Quintero, J.M., Delgado-V., C.A., Palacio, A., Botero, J.M., Múnera, W.A., 2008. Avifauna del norte de la Cordillera Central de los Andes, Colombia. Ornitología Neotropical 19, 495-515.

[80] Socolar, S.J., Robinson, S.K., Terborgh, J., 2013. Bird diversity and occurrence of bamboo specialists in two bamboo die-offs in southeastern Peru. Condor 115, 253-262.

[81] Giraudo, A.R., Matteucci, S.D., Alonso, J., Herrera, J., Abramson, R.R., 2008. Comparing bird assemblages in large and small fragments of the Atlantic Forest hotspots. Biodiversity and Conservation 17, 1251-1265.

[82] Walker, B., Stotz, D.F., Pequeño, T., Fitzpatrick, J.W., 2006. Birds of the Manu Biosphere Reserve. In: Mammals and birds of the Manu Biosphere Reserve, Peru, eds. Patterson, B.D., Stotz, D.F., Solari, S. Field Museum of Natural History, Chicago, Illinois, pp. 23-49.

[83] Goerck, J.M., 1999. Distribution of birds along an elevational gradient in the Atlantic forest of Brazil: implications for the conservation of endemic and endangered species. Bird Conservation International 9, 235-253.

[84] Cardoso da Silva, J.M., Uhl, C., Murray, G., 1996. Plant succession, landscape management, and the ecology of frugivorous birds in abandoned Amazonian pastures. Conservation Biology 10, 491-503.

[85] Vuilleumier, F., 1993. Biogeografía de aves en el neotrópico: jerarquías conceptuales y perspectivas para futuras investigaciones. Revista Chilena de Historia Natural

[86] Vanderhoff, E.N., Grafton, B., 2009. Behavior of tamarins, tanagers and manakins foraging in a strangler fig (Ficus sp.) in Suriname, South America: implications for seed dispersal. Biota Neotropica 9, 419-423.

[87] Robbins, M.B., Braun, M.J., Milensky, C.M., Schmidt, B.K., Prince, W., Rice, N.H., Finch, D.W., O’Shea, B.J., 2007. Avifauna of the upper Essequibo river and Acary mountains, Southern Guyana. Ornitología Neotropical 18, 339-368.

[88] Guix, J.C., 2007. The role of alien plants in the composition of fruit-eating bird assemblages in Brazilian urban ecosystems. Orsis 22, 87-104.

[89] Cohn-Haft, M., Whittaker, A., Stouffer, P.C., 1997. A new look at the "species-poor" Central Amazon: the avifauna north of Manaus, Brazil. Ornithological Monographs 48, 205-235."

[90] Thiollay, J.M., Jullien, M., 1998. Flocking behaviour of foraging birds in a neotropical rain forest and the antipredator defence hypothesis. Ibis 140, 382-394.

[91] Naka, L.N., Cohn-Haft, M., Mallet-Rodrigues, F., Santos, M.P.D., Torres, M.F., 2006. The avifauna of the Brazilian state of Roraima: bird distribution and biogeography in the Rio Branco basin. Revista Brasileira de Ornitologia 14, 197-238.

[92] Banks-Leite, C., Cintra, R., 2008. The heterogeneity of amazonian treefall gaps and bird community composition. Ecotropica 14, 1-13.

[93] de Foresta, H., Charles-Dominique, C., Érard, C., Prévost, M.F., 1984. Zoochorie et premiers stades de la régénération naturelle après coupe en forêt guyanaise. Revue d'Écologie 39, 369-400.

[94] dos Anjos, L., 2001. Bird communities in five Atlantic forest fragments in southern Brazil. Ornitologia Neotropical 12, 11-27.

[95] Sanaiotti, T.M., Cintra, R., 2001. Breeding and migrating birds in an Amazonian savanna. Studies on Neotropical Fauna and Environment 36, 23-32.

[96] Andrade, G.I., Rubio-Torgler, H., 1994. Sustainable use of the tropical rain forest: evidence from the avifauna in a shifting-cultivation habitat mosaic in the Colombian Amazon. Conservation Biology 8, 545-554.

[97] Zurita, G.A., Zuleta, G.A., 2009. Bird use of logging gaps in a subtropical mountain forest: the inﬂuence of habitat structure and resource abundance in the Yungas of Argentina. Forest Ecology and Management 257, 271–279.

[98] Marini, M.A., Motta, J.C. Jr, Vasconcellos, L.A.S., Cavalcanti, R.B., 1997. Avian body masses from the cerrado region of central Brazil. Ornitologia Neotropical 8, 93-99.

[99] Smith, P., Onley, D., Northcote-Smith, E., Atkinson, K., 2012. Morphometrics of cerrado birds from the Reserva Natural Laguna Blanca (NE Paraguay). The Ring 34, 51-67.

[100] Filloy, J., Zurita, G.A., Corbelli, J.M., Bellocq, M.I., 2010. On the similarity among bird communities: testing the inﬂuence of distance and land use. Acta Oecologica 36, 333-338.

[101] Kirwan, G.M., 2009. Notes on the breeding ecology and seasonality of some Brazilian birds. Revista Brasileira de Ornitologia 17, 121-136.

[102] Cisneros-Heredia, D.F., 2006. Notes on breeding, behaviour and distribution of some birds in Ecuador. Bulletin of the British Ornithologists' Club 126, 153-164.

[103] Braun, M.J., Finch, D.W., Robbins, M.B., Schmidt, B.K., 2007. A field checklist of the birds of Guyana, 2nd ed. Smithsonian Institution, Washington, D.C.

[104] de Toledo, M.C.B., Donatelli, R.J., Batista, G.T., 2012. Relation between green spaces and bird community structure in an urban area in Southeast Brazil. Urban Ecosystems 15, 111-131.

[105] Bugoni, L., Mohr, L.V., Scherer, A., Efe, M.A., Barbosa Scherer, S., 2002. Biometry, molt and brood patch paramters of birds in southern Brazil. Ararajuba 10, 85-94.

[106] Suertegaray Fontana, C., Burger, M.I., Magnusson, W.E., 2011. Bird diversity in a subtropical South-American City: effects of noise levels, arborisation and human population density. Urban Ecosystems 14, 341-360.

[107] Marini, M.Â, Borges, F.J.A., Lopes, L.E., Sousa, N.O.M., Gressler, D.T., Santos, L.R., Paiva, L.V., Duca, C., Manica, L.T., Rodrigues, S.S., França, L.F., Costa, P.M., França, L.C., Heming, N.M., Silveira, M.B., Pereira, Z.P., Lobo, Y., Medeiros, R.C.S., Roper, J.J., 2012. Breeding biology of birds in the Cerrado of Central Brazil. Ornitologia Neotropical 23, 385-405.

[108] Morton, E.S., 1976. Vocal mimicry in the thick-billed Euphonia. Wilson Bulletin 88, 485-487.

[109] Ricklefs, R.E., 1977. Reactions of some Panamanian birds to human intrusion at the nest. Condor 79, 376-379.

[110] Munves, J., 1975. Birds of a highland clearing in Cundinamarca, Colombia. Auk 92, 307-321.

[111] Wiersma, P., Muñoz-Garcia, A., Walker, A., Williams, J.B., 2007. Tropical birds have a slow pace of life. Proceedings of the National Academy of Sciences of the United States of America 104, 9340-9345.

[112] Tieleman, B.I., Williams, J.B., 2000. The adjustment of avian metabolic rates and water fluxes to desert environments. Physiological and Biochemical Zoology 73, 461-479.

[113] McKechnie, A.E., Wolf, B.O., 2004. The allometry of avian basal metabolic rate: good predictions need good data. Physiological and Biochemical Zoology 77, 502-521.

[114] Tieleman, B.I., Williams, J.B., Ricklefs, R.E., 2004. Nest attentiveness and egg temperature do not explain the variation in incubation periods in tropical birds. Functional Ecology 18, 571-577.

[115] Robinson, W.D., 1999. Long-term changes in the avifauna of Barro Colorado Island, Panama, a tropical forest isolate. Conservation Biology 13, 85-97.

[116] Nadkarni, N.M., Matelson, T.J., 1989. Bird use of epiphyte resources in neotropical trees. Condor 91, 891-907.

[117] Cardoso da Silva, J.M., 1996. Distribution of Amazonian and Atlantic birds in gallery forests of the Cerrado region, South America. Ornitologia Neotropical 7, 1-18.

[118] Johns, A.D., 1991. Responses of Amazonian rain forest birds to habitat modification. Journal of Tropical Ecology 7, 417-437.

[119] Robinson, W.D., Austin, S.H., Robinson, T.R., Ricklefs, R.E., 2014. Incubation temperature does not explain variation in the embryo development periods in a sample of Neotropical passerine birds. Journal of Ornithology 155, 45-51.

[120] Ridgely, R.S., Tudor, G., 1989. The birds of South America, Volume 1, The oscine passerines. University of Texas Press, Austin.

[121] Karr, J.R., 1971. Ecological, behavioral, and distributional notes on some Central Panamá birds. Condor 73, 107-111.

[122] Wendelken, P.W., Martin, R.F., 1989. Recent data on the distribution of birds in Guatemala, 2. Bulletin of the British Ornithologists' Club 109, 31-36.

[123] Lasky, J.R., Keitt, T.H., 2012. The effect of spatial structure of pasture tree cover on avian frugivores in eastern Amazonia. Biotropica 44, 489-497.

[124] Boyle, W.A., 2011. Short-distance partial migration of Neotropical birds: a community-level test of the foraging limitation hypothesis. Oikos 120: 1803-1816.

[125] Thiollay, J.M., 1994. Structure, density and rarity in an Amazonian rain forest bird community. Journal of Tropical Ecology 10: 449-481.

[126] Gleffe, J.D., Collazo, J.A., Groom, M.J., Miranda-Castro, L., 2006. Avian reproduction and the conservation value of shaded coffee plantations. Ornitologia Neotropical 17, 271–282.

[127] Schmitt, C.G., Schmitt, D.C., Remsen, J.V. Jr, 1997. Birds of the Tambo area, an arid valley in the Bolivian Andes. Ornithological Monographs 48, 701-716.

[128] Latta, S.C., Wunderle, J.M. Jr, 1996. The composition and foraging ecology of mixed-species flocks in pine forests of Hispaniola. Condor 98, 595-607.

[129] Faaborg, J., 1977. Metabolic rates, resources, and the occurrence of nonpasserines in terrestrial avian communities. American Naturalist 111, 903-916.

[130] Holland, C.S., Williams, J.M., 1978. Observations on the birds of Antigua. American Birds 32, 1095-1105.

[131] Graves, G.R., 1985. Elevationall correlates of speciation and intraspecific geographic variation in plumage in Andean forest birds. Auk 102, 556-579.

[132] Walker, B., Stotz, D.F., Pequeño, T., Fitzpatrick, J.W., 2006. Birds of the Manu biosphere reserve. Fieldiana, Zoology 110, 23-49.

[133] Borges, S.H., 2006. Rarity of birds in the Jaú National Park, Brazilian Amazon. Animal Biodiversity and Conservation 29, 179-189.

[134] Crease, A., 2008. Avian range extensions from the southern headwaters of the río Caroní, Gran Sabana, Bolívar, Venezuela. Cotinga 31, 5-19.

[135] Vidoz, J.Q., Jahn, A.E., Mamani, A.M., 2010. The avifauna of Estación Biológica Caparú, Bolivia. Cotinga 32, 51-68.

[136] Socolar, S.J., González, Ó, Forero-Medina, G., 2013. Noteworthy bird records from the northern Cerros del Sira, Peru. Cotinga 35, 24-36.

[137] Mestre, L.A.M., Thom, G., Cochrane, M.A., Barlow, J., 2010. The birds of reserva extrativista Chico Mendes, South Acre, Brazil. Boletim do Museu Paraense Emílio Goeldi, Ciências Naturais 5, 311-333.

[138] Henriques, L.M.P., Wunderle, J.M. Jr., Willig, M.R., 2003. Birds of the Tapajos national forest, Brazilian Amazon: a preliminary assessment. Ornitologia Neotropical 14, 307-338.

[139] Johnston, J.P., Peach, W.J., Gregory, R.D., White, S.A., 1997. Survival rates of tropical and temperate passerines: a Trinidadian perspective. American Naturalist 150, 771-789.

[140] Snow, B.K., Snow, D.W., 1971. The feeding ecology of tanagers and honeycreepers in Trinidad. Auk 88, 291-322.

[141] del Hoyo, J., Elliott, A., Christie, D.A., 2010. Handbook of the birds of the world, Volume 15, Weavers to New World warblers. Lynx Edicions, Barcelona.

[142] del Hoyo, J., Elliott, A., Christie, D.A., 2011. Handbook of the birds of the world, Volume 16, Tanagers to New World blackbirds. Lynx Edicions, Barcelona.

[143] Isler, M.L., Isler, P.R., 1987. The tanagers: natural history, distribution, and identification. Smithsonian Institution Press, Washington, D.C.

[144] <https://pt.wikipedia.org/wiki/>

[145] <http://www.birdlife.org/datazone/species>

[146] <https://de.wikipedia.org/wiki/>

[147] <https://fr.wikipedia.org/wiki/>

[148] <https://es.wikipedia.org/wiki/>

[149] <https://en.wikipedia.org/wiki/>

[150] <http://genomics.senescence.info/species/browser.php?type=4&name=Fringillidae>

[151] Bennett, P.M., Harvey, P.H., 1987. Active and resting metabolism in birds: allometry, phylogeny and ecology. Journal of Zoology 213, 327-363.

[152] Van Riper, C III, 1984. The influence of nectar resources on nesting success and movement patterns of the Common Amakihi (Hemignathus virens). Auk 101, 38-46.

[153] Pletschet, S.M., Kelly, J.F., 1990. Breeding biology and nesting success of palila. Condor 92, 1012-1021.
