# Supplementary material for: Ancestrality and evolution of trait syndromes in finches (Fringillidae)
Source: Ecol Evol. 2017 Oct 21;7(23):9935–53. doi: 10.1002/ece3.3420 (PMC5723631; doi:10.1002/ece3.3420)
Supplement: Supplementary file 4 [file ECE3-7-9935-s004.pdf]

Appendix S4. Supermatrix tree of Fringillidae for three nuclear introns and five mitochondrial genes.

S 4.1. Assembling the supermatrix

We downloaded all Fringillidae sequences in Genbank and sorted them by taxon and locus. After counting how many taxa were available per locus, we decided to include in the supermatrix three introns and five mitochondrial genes (see locus list below), all of which were represented by at least 50 species. For each sequence, length, associated specimen/sample number and locality were retrieved from Genbank and/or the corresponding publication and combined in a preliminary table. Possible misidentification or anomalous sequences were checked using simple neighbor-joining tree.

For each species we selected one sequence per locus. Whenever possible, for a given species we selected the sequences originated from the same specimen or minimized the number of specimens, and we tried also to choose sequences obtained from the same subspecies or with similar geographic origin.

Table S 4.1. Genes, alignment length and number of sequence per gene included in the supermatrix.

Locus	Alignment length (bp)	Number of sequences
Glyceraldehyde-3-phosphate dehydrogenase (GAPDH), intron 11	327	116 (68.6 %)
Myoglobin gene, intron 2	741	116 (68.69 %)
Ornithine decarboxylase (ODC) gene, introns 6-7	697	112 (66. %)
Cytochrome b (cytb)	1143	129 (76.3 %)
NADH dehydrogenase subunit II (ND2)	1041	133 (78.7 %)
NADH dehydrogenase subunit III (ND3)	351	129 (76.3 %)
Cytochrome oxidase subunit I (COI)	648	59 (34.9 %)
ATPase subunit 6 (ATP6)	684	81 (47.9 %)

Table S 4.2. Species and Genbank accession numbers included in the supermatrix.

Taxon	Specimen	GAPDH	Myoglobin	ODC	cytb	ND2	ND3	COI	ATP6
Fringilla coelebs	NRM 956301	JN715183	JN715273	JN715365		JN715455	JN715548		
Fringilla teydea	NHMO				KU705760	KU705760	KU705760	KU705760	KU705760
Fringilla montifringilla	NRM 20046395	JN715184	GU816941	GU816920		GU816851	GU816816		
Chlorophonia cyanea	NRM 20066989	JN715171	JN715263	JN715353			JN715537		
Chlorophonia cyanea	USNM 625305							JQ174424	
Chlorophonia occipitalis	no information					EF529844			
Chlorophonia occipitalis	no information				EF529956				
Euphonia plumbea	USNM 632543							JQ174819	
Euphonia affinis	529-05							EU442311	
Euphonia affinis	no information				EU442360				
Euphonia luteicapilla	USNM 612448							JQ174817	
Euphonia chlorotica	NRM 956750	JN715175	AY228298	JN715357		JN715448	JN715541		
Euphonia finschi	FMNH 389276				AF290143				
Euphonia finschi	NRM 20066306	JN715180	JN715270	JN715362			JN715545		
Euphonia violacea	NRM 966943	JN715181	JN715271	JN715363		JN715453	JN715546		
Euphonia laniirostris	LSU B18375				AF006232				
Euphonia laniirostris	LSU B18411								AF447330
Euphonia laniirostris	NRM 20066309	JN715176	JN715266	JN715358		JN715449			
Euphonia hirundinacea	528-15							EU442301	
Euphonia hirundinacea	no information				EU442333				
Euphonia elegantissima	KU 87070							JQ174807	

Euphonia cyanocephala	MACN-Or-ct 913							FJ027571	
Euphonia musica	no information				AF310067				
Euphonia musica	NRM 976696	JN715178	JN715268	JN715360		JN715451	JN715543		
Euphonia fulvicrissa	STRI PAEFU102				AF383014	AF383130			AF382975
Euphonia gouldi	CU 44255					FJ231698			
Euphonia chrysopasta	KU 89079							JQ174806	
Euphonia minuta	NRM 20066307	JN715177	JN715267	JN715359		JN715450	JN715542		
Euphonia anneae	USNM 607624							JQ174801	
Euphonia xanthogaster	NRM 20066305	JN715182	JN715272	JN715364		JN715454	JN715547		
Euphonia rufiventris	NRM 20066310	JN715179	JN715269	JN715361		JN715452	JN715544		
Euphonia pectoralis	MACN-Or-ct 2867							FJ027574	
Euphonia cayennensis	NRM 20056062	JN715174	JN715265	JN715356			JN715540		
Mycerobas icterioides	FMNH 256357				KJ456356				
Mycerobas affinis	AMNH 5592				KJ456354				
Mycerobas melanozanthos	FMNH 222122				KJ456357				
Mycerobas carnipes	NRM 23363	JN715196	JN715284	JN715376		JN715466	JN715558		
Hesperiphona vespertina	NRM 23336	JN715186	JN715275	JN715367		JN715457	JN715550		
Hesperiphona vespertina	UMMZ 235311				KM078770			KM078770	KM078770
Coccothraustes coccothraustes	NRM 976374	JN715172	AY228292	JN715354	AY228055	JN715446	JN715538	GU571828	
Eophona migratoria	no information				AF342871				
Eophona migratoria	NRM 896473	JN715173	JN715264	JN715355		JN715447	JN715539		
Eophona personata	no information				AF342872				
Melamprosops phaeosoma	BPBM 147112	KM112521		KM112827	KM078793	KM078793	KM078793	KM078793	KM078793
Paroreomyza montana	NZP USFWS band 2141-67177				KM078771			KM078771	KM078771

Paroreomyza montana	RCF 1984	JN715197	JN715285	JN715377		JN715467	JN715559		
Dysmorodrepanis munroi	NZP USFWS band 1581-75367	KM112533		KM112815	KM078774	KM078774	KM078774	KM078774	KM078774
Telespiza cantans	NZP USFWS band 8061-85532	KM112508		KM112859	KM078777	KM078777	KM078777	KM078777	KM078777
Telespiza ultima	Conant 92.7.26-1	KM112509		KM112811	KM078787	KM078787	KM078787		KM078787
Loxioides bailleui	MRSNT 5783	JN715195	JN715283	JN715375		JN715465	JN715557		
Loxioides bailleui	NZP USFWS band 8031-75805							KM078805	KM078805
Psittirostra psittacea	no information				KU158196	KU158196	KU158196	KU158196	KU158196
Oreomystis bairdi	MVZ 178406	KM112527	KC007674	KM112853	KM078807	KM078807	KM078807	KM078807	KM078807
Magumma parva	MVZ 178407	KM112536		KM112805	KM078799	KM078799	KM078799	KM078799	KM078799
Loxops caeruleirostris	MVZ 178405	KM112505		KM112834	KM078776	KM078776	KM078776	KM078776	KM078776
Loxops coccineus	NZP USFWS band 2350-43262	KM112523		KM112807	KM078785	KM078785	KM078785	KM078785	KM078785
Manucerthia mana	TKP 559	KM112532		KM112820	KM078768	KM078768	KM078768	KM078768	KM078768
Chlorodrepanis virens	NZP RCF-3425				KM078788			KM078788	KM078788
Chlorodrepanis virens	RCF 2913	JN715225	JN715313	JN715405		JN715496	JN715588		
Chlorodrepanis flava	NZP USFWS band 1680-10360	KM112537		KM112821	KM078780	KM078780	KM078780	KM078780	KM078780
Chlorodrepanis stejnegeri	NZP RCF-2666	KM112512		KM112809	KM078801	KM078801	KM078801	KM078801	KM078801
Hemignathus wilsoni	NZP RCF-3422	KM112535		KM112823	KM078802	KM078802	KM078802	KM078802	KM078802
Akialoa obscura	no information				KU158190	KU158190	KU158190	KU158190	KU158190
Pseudonestor xanthophrys	NZP USFWS band 8051-40968	KM112541		KM112840	KM078809	KM078809	KM078809	KM078809	KM078809
Vestiaria coccinea	NZP USFWS band 1471-19056	KM112546		KM112847	KM078797	KM078797	KM078797	KM078797	KM078797
Himatione sanguinea	NZP RCF-3427	KM112501		KM112808	KM078773	KM078773	KM078773	KM078773	KM078773
Palmeria dolei	BPBM 184556	KM112499		KM112801	KM078803	KM078803			
Erythrina erythrina	NRM 976373	JN715154	JN715246	JN715336		JN715428	JN715519	GU571800	
Erythrina erythrina	USNM B18937				KM078766			KM078766	KM078766

Haematospiza sipahi	no information				AF342875				
Haematospiza sipahi	NRM 23812	JN715185	JN715274	JN715366		JN715456	JN715549		
Chaunoproctus ferreorostris	BMNH 1855.12.19.71					JN715445	JN715536		
Carpodacus synoicus	MAR 5084				KF194098				
Carpodacus synoicus	NHMO 26633	JN715168	JN715260	JN715350		JN715442	JN715533		
Carpodacus stoliczkae	BMNH 1967.17803		KF194009		KF194103	KF194147			
Carpodacus roborowskii	NRM 23882	JN715161	JN715253	JN715343		JN715435	JN715526		
Carpodacus rubicilloides	MAR 5931				KF194089				
Carpodacus rubicilloides	NRM 23864	JN715167	JN715259	JN715349		JN715441	JN715532		
Carpodacus rubicilla	NRM 20016594	JN715166	JN715258	JN715348		JN715440	JN715531		
Carpodacus rubicilla	ZFMK J.II.22.f.l				KF194088				
Carpodacus sibiricus	NRM 20076294	JN715224	JN715312	JN715404		JN715495	JN715587		
Carpodacus sibiricus	UWBM 47588				KM078763			KM078763	KM078763
Carpodacus puniceus	FMNH 277071				KF194173				
Carpodacus puniceus	NRM 23927	JN715158	JN715250	JN715340		JN715432	JN715523		
Carpodacus subhimachalus	FMNH 277061				KF194180				
Carpodacus subhimachalus	NRM 23477	JN715199	JN715287	JN715379		JN715469	JN715561		
Carpodacus roseus	NRM 20026495	JN715164	JN715256	JN715346		JN715438	JN715529		
Carpodacus roseus	USNM B10230				KM078779			KM078779	KM078779
Carpodacus trifasciatus	MTD C63834	KF194004	KF194019		KF194115	KF194166			
Carpodacus thura	AMNH DOT5609		KF194034	KJ455736	KF194181	KF194135			
Carpodacus dubius	MAR 369				KF194106				
Carpodacus dubius	NRM 20016581	JN715169	JN715261	JN715351		JN715443	JN715534		
Carpodacus rhodochlamys	NRM 20026491	JN715160	JN715252	JN715342	KF194174	JN715434	JN715525		

Carpodacus grandis	ZFMK J.II.22.g1.g		KF194010		KF194051	KF194152			
Carpodacus davidianus	MTD C64235	KF193991	KF194018		KF194060	KF194156			
Carpodacus pulcherrimus	NRM 20026494	JN715157	JN715249	JN715339	KF194172	JN715431	JN715522		
Carpodacus waltoni	MAR 5936	KF193993	KF194017		KF194057	KF194141			
Carpodacus edwardsii	MTD C24119				KF194039				
Carpodacus verreauxii	CAS 95886		KF194033		KF194169	KF194128			
Carpodacus verreauxii	MNHN 19-37								EU880942
Carpodacus rodochroa	AMNH DOT5671				KF194176				
Carpodacus rodochroa	NRM 553889	JN715162	JN715254	JN715344		JN715436	JN715527		
Carpodacus rodopeplus	RMNH 44517	JN715163	JN715255	JN715345		JN715437	JN715528		
Carpodacus formosanus	NMNST 7838	KF193996	KF194006		KF194119	KF194167			
Carpodacus vinaceus	MNHN 19.23								EU880943
Carpodacus vinaceus	NRM 20026493	JN715170	JN715262	JN715352		JN715444	JN715535		
Carpodacus vinaceus	U Mainz MAR 961				HQ284695				
Pinicola enucleator	NRM 996174	JN715198	JN715286	JN715378	HQ284684	JN715468	JN715560	GU572046	
Pyrrhula nipalensis	MAR ES11				KF194184				
Pyrrhula nipalensis	NRM 23476	JN715202	JN715290	JN715382		JN715472	JN715564		
Pyrrhula leucogenis	CMC 36632	HQ284782			HQ284678				
Pyrrhula aurantiaca	BMNH 1949.25.3775				HQ284579				
Pyrrhula erythrocephala	MNHN 18-05		EU878710				EU881015		EU880949
Pyrrhula erythrocephala	U Mainz MAR 90021				HQ284642				
Pyrrhula erythaca	NRM 20016568	JN715201	JN715289	JN715381	HQ284652	JN715471	JN715563		
Pyrrhula murina	U Sheffield TRB2	HQ284768	KX109679	KX109755	HQ284631	KX109700			
Pyrrhula pyrrhula	MNHN 95-25								EU880950

Pyrrhula pyrrhula	NRM 20046541	JN715203	JN715291	JN715383		JN715473	JN715565	GU572069	
Pyrrhula pyrrhula	U Mainz MAR 4437				HQ284595				
Rhodopechys sanguineus	NRM 20026504	JN715207	JN715295	JN715387		JN715477	JN715569		
Bucanetes githagineus	MTD C64524 MAR1172				HQ284701				
Bucanetes githagineus	NRM 20046702	JN715204	JN715292	JN715384		JN715474	JN715566		
Eremopsaltria mongolica	NRM 23495	JN715205	JN715293	JN715385		JN715475	JN715567		
Eremopsaltria mongolica	USNM B10185				KM078792			KM078792	KM078792
Agraphospiza rubescens	MART 90161				KF194120				
Agraphospiza rubescens	NRM 23826	JN715165	JN715257	JN715347		JN715439	JN715530		
Callacanthis burtoni	NRM 24869	JN715135	JN715227	JN715317		JN715409	JN715500		
Pyrrhoplectes epauletta	MNHN 16-2j								EU880948
Pyrrhoplectes epauletta	MTD C63109 MAR5549				HQ284689				
Pyrrhoplectes epauletta	NRM 23810	JN715200	JN715288	JN715380		JN715470	JN715562		
Procarduelis nipalensis	no information				AF342866				
Procarduelis nipalensis	NRM 23824	JN715156	JN715248	JN715338		JN715430	JN715521		
Leucosticte nemoricola	IZAS uncat	JN715189	JN715278	JN715370		JN715460	JN715553		
Leucosticte nemoricola	MTD C62013 MAR4246				HQ284700				
Leucosticte brandti	NRM 24575	JN715188	JN715277	JN715369		JN715459	JN715552		
Leucosticte brandti	UWBM 66356				KM078775			KM078775	KM078775
Leucosticte sillemi	ZMA.AVES 43449					KT444635			
Leucosticte arctoa	NRM 24559	JN715187	JN715276	JN715368		JN715458	JN715551		
Leucosticte arctoa	USNM 609762				KM078791			KM078791	KM078791
Leucosticte tephrocotis	NRM 20016579	JN715190	JN715279	JN715371		JN715461			
Leucosticte tephrocotis	UWBM 66944 SVD 2371				KX109627				

Haemorhous mexicanus	NRM 20056140	JN715155	JN715247	JN715337		JN715429	JN715520		
Haemorhous mexicanus	NZP RCF-2645				KM078782			KM078782	KM078782
Haemorhous cassinii	USNM B20075	KM112518		KM112813	KM078786	KM078786	KM078786	KM078786	KM078786
Haemorhous purpureus	NRM 557743	JN715159	JN715251	JN715341		JN715433	JN715524		
Haemorhous purpureus	UMMZ 227048 T-126				KM078772				KM078772
Rhodospiza obsoleta	NRM 20046707	JN715206	JN715294	JN715386		JN715476	JN715568		
Rhynchostruthus socotranus	NRM 23483	JN715208	JN715296	JN715388		JN715478	JN715570		
Chloris chloris	MNHN C34								EU880931
Chloris chloris	NRM 986328	JN715141	JN715233	JN715323		JN715415	JN715506	GU571791	
Chloris chloris	SMTD C 62175				HQ284692				
Chloris sinica	NRM 20026538	JN715150	JN715242	JN715332		JN715424	JN715515		
Chloris sinica	USNM B19440				KM078783			KM078783	KM078783
Chloris spinoides	NRM 20026503	JN715151	JN715243	JN715333	KJ456211	JN715425	JN715516		
Chloris spinoides	ZMUC 131646								EU880974
Chloris monguilloti	NRM 546196	JN715147	JN715239	JN715329		JN715421	JN715512		
Chloris ambigua	no information				U78322				
Chloris ambigua	NRM 20026539	JN715136	JN715228	JN715318		JN715410	JN715501		
Chloris ambigua	ZMUC 118651								EU880992
Linurgus olivaceus	MNHN 40-23								EU880938
Linurgus olivaceus	NRM 20086232	JN715191	JN715280	JN715372		JN715462	JN715554		
Crithagra citrinelloides	no information				AY790888				
Crithagra citrinelloides	NRM 20026501	JN715212	JN715300	JN715392		JN715482	JN715574		
Crithagra citrinelloides	ZMUC 134740								EU880968
Crithagra hyposticta	ZMUC 131127		EU878731				EU881039		EU880971

Crithagra capistrata	ZMUC 135500		EU878742				EU881052		EU880984
Crithagra scotops	no information				AY790894				
Crithagra leucopygia	no information				L76264				
Crithagra leucopygia	NRM 20106050	JN715214	JN715302	JN715394		JN715485	JN715577		
Crithagra leucopygia	ZMUC 118662								EU880993
Crithagra atrogularis	no information				L76267				
Crithagra citrinipectus	ZMUC 130559		EU878717				EU881025		EU880957
Crithagra mozambica	MNHN 40-54								EU880953
Crithagra mozambica	no information				L76265				
Crithagra mozambica	NRM 20066026	JN715216	JN715304	JN715396		JN715487	JN715579		
Crithagra flaviventris	no information				AY790887				
Crithagra flaviventris	ZMUC 132129		EU878732				EU881040		EU880972
Crithagra dorsostriata	UMMZ 233806 T-810c	KM112547		KM112837	KM078798	KM078798	KM078798	KM078798	KM078798
Crithagra sulphurata	no information				AY790889				
Crithagra sulphurata	NRM 20026498	JN715221	JN715309	JN715401		JN715492	JN715584		
Crithagra albogularis	UWBM 70375	KM112520		KM112836	KM078764	KM078764	KM078764	KM078764	KM078764
Crithagra reichardi	ZMUC 122436		EU878734				EU881043		EU880975
Crithagra gularis	no information				L77556				
Crithagra gularis	ZMUC 131650						EU881041		EU880973
Crithagra mennelli	NRM 20026500	JN715215	JN715303	JN715395		JN715486	JN715578		
Crithagra mennelli	ZMUC 118671								EU880990
Crithagra striolata	no information				AY790895				
Crithagra striolata	NRM 23718	JN715220	JN715308	JN715400		JN715491	JN715583		
Crithagra striolata	ZMUC 134756								EU880969

Crithagra burtoni	no information				AY790896				
Crithagra burtoni	NRM 20086267	JN715209	JN715297	JN715389		JN715479	JN715571		
Crithagra burtoni	ZMUC 123647								EU880997
Crithagra melanochroa	ZMUC 118856		EU878737				EU881046		EU880978
Crithagra rufobrunnea	NRM 857618	JN715218	JN715306	JN715398		JN715489	JN715581		
Crithagra totta	no information				AY790892				
Linaria flavirostris	JM136				KJ456172				
Linaria flavirostris	NRM 20066634	JN715144	JN715236	JN715326		JN715418	JN715509	GU571794	
Linaria cannabina	MNHN 25-82								EU880939
Linaria cannabina	NRM 966403	JN715139	JN715231	JN715321		JN715413	JN715504	GU571787	
Linaria cannabina	SMTD C 61033				HQ284691				
Acanthis flammea	no information				L76386				
Acanthis flammea	NRM 20016449	JN715143	JN715235	JN715325		JN715417	JN715508	GU571793	
Acanthis hornemanni	AJN 000043	JN715145	JN715237	JN715327		JN715419	JN715510	GU571795	
Acanthis hornemanni	no information				U83201				
Acanthis hornemanni	ZMUC 119818								EU880989
Loxia pytyopsittacus	NRM 20046001	JN715194	JN715282	JN715374		JN715464	JN715556	GU571962	
Loxia pytyopsittacus	ZMUC 119824								EU880986
Loxia scotica	no information				AF171656				
Loxia curvirostra	NRM 976546	JN715192	AY228303	GU816921	AY228065	GU816852	GU816817	GU571958	
Loxia leucoptera	no information				AF342878				
Loxia leucoptera	NRM 20026565	JN715193	JN715281	JN715373		JN715463	JN715555	GU571960	
Loxia leucoptera	ZMUC 116705								EU880962
Chrysocorythus estherae	RMNH 44712					JN715483	JN715575		

Carduelis carduelis	NRM 996076	JN715140	JN715232	JN715322		JN715414	JN715505	GU571789	
Carduelis carduelis	NZP USFWS band 1760-76299							KM078790	KM078790
Carduelis citrinella	MNHN C38								EU880933
Carduelis citrinella	no information				L77872				
Carduelis citrinella	NRM 553307	JN715211	JN715299	JN715391		JN715481	JN715573		
Serinus serinus	no information				L76263				
Serinus serinus	NRM 20046491	JN715219	JN715307	JN715399		JN715490	JN715582	GU572089	
Serinus serinus	ZMUC 116715								EU880980
Serinus canaria	NRM 20026502	JN715213	JN715301	JN715393		JN715484	JN715576		
Serinus canaria	NZP 105				KM078794			KM078794	KM078794
Serinus syriacus	NRM 23600	JN715222	JN715310	JN715402		JN715493	JN715585		
Serinus pusillus	JM128				KJ456465				
Serinus pusillus	NRM 20046715	JN715217	JN715305	JN715397		JN715488	JN715580		
Serinus pusillus	ZMUC 135626								EU880983
Serinus alario	no information				AY790899				
Serinus canicollis	no information				AY790890				
Serinus canicollis	NRM 20076189	JN715210	JN715298	JN715390		JN715480	JN715572		
Serinus canicollis	ZMUC 128624								EU880958
Serinus flavivertex	no information				AY790897				
Spinus thibetanus	BMNH 1948.34.64	JN715223	JN715311	JN715403		JN715494	JN715586		
Spinus thibetanus	no information				L76279				
Spinus spinus	MNHN 2000.1651								EU880941
Spinus spinus	no information				L76391				
Spinus spinus	NRM 986184	JN715152	JN715244	JN715334		JN715426	JN715517	GU571799	

Spinus pinus	NRM 20016375	JN715148	JN715240	JN715330		JN715422	JN715513		
Spinus pinus	UMMZ 227858				KM078796			KM078796	KM078796
Spinus tristis	MSB 29161				KT221304				
Spinus tristis	NRM 20016378	JN715153	JN715245	JN715335		JN715427	JN715518		
Spinus psaltria	NRM 20016376	JN715149	JN715241	JN715331		JN715423	JN715514		
Spinus psaltria	NZP USFWS band 2120-99480				KM078806			KM078806	KM078806
Spinus lawrencei	MVZ 176985		KT221368		KT221302	KT221172			
Spinus atriceps	01N6050								KT358782
Spinus atriceps	AMNH DOT7151		KT221375		KT221309	KT221179	KT221243		
Spinus spinescens	ANSP 18807				KT221360	KT221230	KT221294		
Spinus spinescens	ZMUC 120509								EU880985
Spinus yarrellii	no information				U83200				
Spinus cucullatus	NRM 20026508	JN715142	JN715234	JN715324		JN715416	JN715507		
Spinus cucullatus	USNM B12867				KT221318				
Spinus cucullatus	ZMUC 135619								EU880982
Spinus crassirostris	MSB 34207		KT221383		KT221317	KT221187	KT221251		
Spinus crassirostris	ZMUC 129671								EU880959
Spinus magellanicus	FMNH 334723				KT221349				
Spinus magellanicus	NRM 986696	JN715146	JN715238	JN715328		JN715420	JN715511		
Spinus magellanicus	ZMUC 120822								EU880988
Spinus dominicensis	AMNH DOT6930		KT221387		KT221322	KT221192	KT221256		
Spinus siemiradzkii	ANSP 19701				KT221358				
Spinus siemiradzkii	ZMUC 116681		EU878725				EU881033		EU880965
Spinus olivaceus	ANSP 19746				KT221354	KT221224	KT221288		

Spinus notatus	03N1083								KT358784
Spinus notatus	FMNH 393899		KT221417		KT221352	KT221222	KT221286		
Spinus xanthogastrus	ANSP 18543				KT221365				
Spinus xanthogastrus	ZMUC 116687		EU878721				EU881029		EU880961
Spinus atratus	MSB 34123				KT221305				
Spinus atratus	NRM 546071	JN715137	JN715229	JN715319		JN715411	JN715502		
Spinus atratus	ZMUC 118878								EU880994
Spinus uropygialis	MSB 33471		KT221422		KT221364	KT221234	KT221298		
Spinus uropygialis	ZMUC 116685								EU880964
Spinus barbatus	AMNH DOT10430				KT221310				
Spinus barbatus	NRM 546142	JN715138	JN715230	JN715320		JN715412	JN715503		

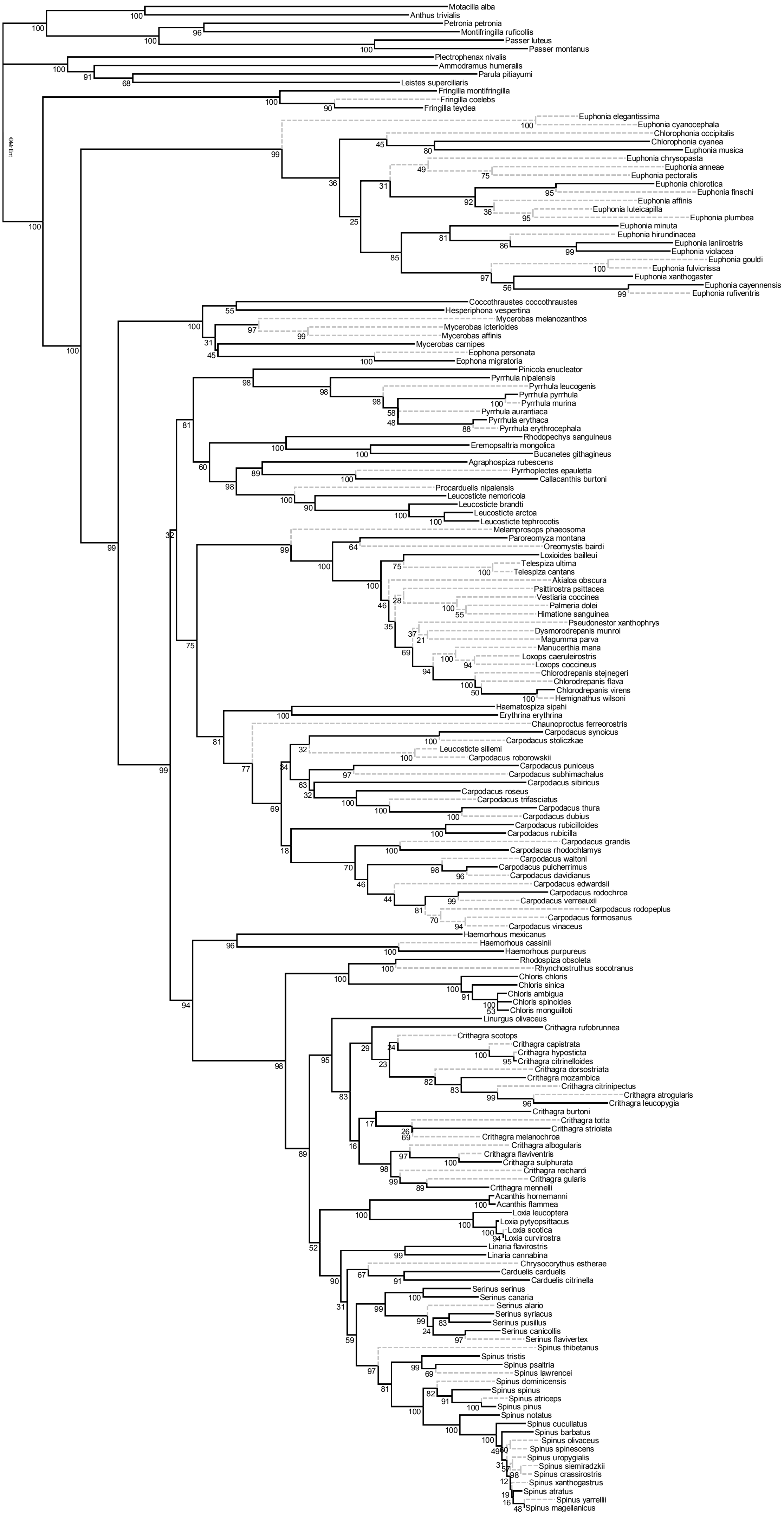
Motacilla alba
Anthus trivialis
Petronia petronia
Montifringilla ruficollis
Passer luteus
Passer montanus
Plectrophenax nivalis
Ammodramus humeralis
Parula pitiayumi
Leistes superciliaris
Fringilla montifringilla
Fringilla coelebs
Fringilla teydea
Euphonia elegantissima
Euphonia cyanocephala
Chlorophonia occipitalis
Chlorophonia cyanea
Euphonia musica
Euphonia chrysopasta
Euphonia anneae
Euphonia pectoralis
Euphonia chlorotica
Euphonia finschi
Euphonia affinis
Euphonia luteicapilla
Euphonia plumbea
Euphonia minuta
Euphonia hirundinacea
Euphonia laniirostris
Euphonia violacea
Euphonia gouldi
Euphonia fulvicrissa
Euphonia xanthogaster
Euphonia cayennensis
Euphonia rufiventris
Coccothraustes coccothraustes
Hesperiphona vespertina
Mycerobas melanozanthos
Mycerobas icterioides
Mycerobas affinis
Mycerobas carnipes
Eophona personata
Eophona migratoria
Pinicola enucleator
Pyrrhula nipalensis
Pyrrhula leucogenis
Pyrrhula pyrrhula
Pyrrhula murina
Pyrrhula aurantiaca
Pyrrhula erythaca
Pyrrhula erythrocephala
Rhodopechys sanguineus
Eremopsaltria mongolica
Bucanetes githagineus
Agraphospiza rubescens
Pyrrhoplectes epauletta
Callacanthis burtoni
Procarduelis nipalensis
Leucosticte nemoricola
Leucosticte brandti
Leucosticte arctoa
Leucosticte tephrocotis
Melamprosops phaeosoma
Paroreomyza montana
Oreomystis bairdi
Loxioides bailleui
Telespiza ultima
Telespiza cantans
Akialoa obscura
Psittirostra psittacea
Vestiaria coccinea
Palmeria dolei
Himatione sanguinea
Pseudonestor xanthophrys
Dysmorodrepanis munroi
Magumma parva
Manucerthia mana
Loxops caeruleirostris
Loxops coccineus
Chlorodrepanis stejnegeri
Chlorodrepanis flava
Chlorodrepanis virens
Hemignathus wilsoni
Haematospiza sipahi
Erythrina erythrina
Chaunoproctus ferreorostris
Carpodacus synoicus
Carpodacus stoliczkae
Leucosticte sillemi
Carpodacus roborowskii
Carpodacus puniceus
Carpodacus subhimachalus
Carpodacus sibiricus
Carpodacus roseus
Carpodacus trifasciatus
Carpodacus thura
Carpodacus dubius
Carpodacus rubicilloides
Carpodacus rubicilla
Carpodacus grandis
Carpodacus rhodochlamys
Carpodacus waltoni
Carpodacus pulcherrimus
Carpodacus davidianus
Carpodacus edwardsii
Carpodacus rodochroa
Carpodacus verreauxii
Carpodacus rodopeplus
Carpodacus formosanus
Carpodacus vinaceus
Haemorhous mexicanus
Haemorhous cassinii
Haemorhous purpureus
Rhodospiza obsoleta
Rhynchostruthus socotranus
Chloris chloris
Chloris sinica
Chloris ambigua
Chloris spinoides
Chloris monguilloti
Linurgus olivaceus
Crithagra rufobrunnea
Crithagra scotops
Crithagra capistrata
Crithagra hyposticta
Crithagra citrinelloides
Crithagra dorsostriata
Crithagra mozambica
Crithagra citrinipectus
Crithagra atrogularis
Crithagra leucopygia
Crithagra burtoni
Crithagra totta
Crithagra striolata
Crithagra melanochroa
Crithagra albogularis
Crithagra flaviventris
Crithagra sulphurata
Crithagra reichardi
Crithagra gularis
Crithagra mennelli
Acanthis hornemanni
Acanthis flammea
Loxia leucoptera
Loxia pytyopsittacus
Loxia scotica
Loxia curvirostra
Linaria flavirostris
Linaria cannabina
Chrysocorythus estherae
Carduelis carduelis
Carduelis citrinella
Serinus serinus
Serinus canaria
Serinus alario
Serinus syriacus
Serinus pusillus
Serinus canicollis
Serinus flavivertex
Spinus thibetanus
Spinus tristis
Spinus psaltria
Spinus lawrencei
Spinus dominicensis
Spinus spinus
Spinus atriceps
Spinus pinus
Spinus notatus
Spinus cucullatus
Spinus barbatus
Spinus olivaceus
Spinus spinescens
Spinus uropygialis
Spinus siemiradzkii
Spinus crassirostris
Spinus xanthogastrus
Spinus atratus
Spinus yarrellii
Spinus magellanicus
